# Psychosocial interventions for improving engagement in care and health and behavioural outcomes for adolescents and young people living with HIV: a systematic review and meta‐analysis

**DOI:** 10.1002/jia2.25741

**Published:** 2021-08-02

**Authors:** Christina A Laurenzi, Stefani du Toit, Wole Ameyan, GJ Melendez‐Torres, Tashmira Kara, Amanda Brand, Yeukai Chideya, Nina Abrahams, Melissa Bradshaw, Daniel T Page, Nathan Ford, Nadia A Sam‐Agudu, Daniella Mark, Marco Vitoria, Martina Penazzato, Nicola Willis, Alice Armstrong, Sarah Skeen

**Affiliations:** ^1^ Institute for Life Course Health Research Department of Global Health Faculty of Medicine and Health Sciences Stellenbosch University Tygerberg South Africa; ^2^ Global HIV, Hepatitis and Sexually Transmitted Infections Programmes World Health Organization Geneva Switzerland; ^3^ Peninsula Technology Assessment Group University of Exeter Exeter United Kingdom; ^4^ Division of Epidemiology and Biostatistics Department of Global Health Faculty of Medicine and Health Sciences Centre for Evidence‐Based Health Care Stellenbosch University Tygerberg South Africa; ^5^ Pediatric and Adolescent Unit Prevention, Care and Treatment Department Institute of Human Virology Nigeria Abuja Nigeria; ^6^ Institute of Human Virology and Department of Pediatrics University of Maryland School of Medicine Baltimore MD USA; ^7^ Paediatric Adolescent Treatment Africa Cape Town South Africa; ^8^ Africaid Harare Zimbabwe; ^9^ UNICEF Eastern and Southern Africa Regional Office Nairobi Kenya

**Keywords:** adolescent HIV, adolescents and young people, psychosocial interventions, adherence to ART, viral load, viral suppression, sexual risk behaviour, engagement in care

## Abstract

**Introduction:**

Adolescents and young people comprise a growing proportion of new HIV infections globally, yet current approaches do not effectively engage this group, and adolescent HIV‐related outcomes are the poorest among all age groups. Providing psychosocial interventions incorporating psychological, social, and/or behavioural approaches offer a potential pathway to improve engagement in care and health and behavioural outcomes among adolescents and young people living with HIV (AYPLHIV).

**Methods:**

A systematic search of all peer‐reviewed papers published between January 2000 and July 2020 was conducted through four electronic databases (Cochrane Library, PsycINFO, PubMed and Scopus). We included randomized controlled trials evaluating psychosocial interventions aimed at improving engagement in care and health and behavioural outcomes of AYPLHIV aged 10 to 24 years.

**Results and discussion:**

Thirty relevant studies were identified. Studies took place in the United States (n = 18, 60%), sub‐Saharan Africa (Nigeria, South Africa, Uganda, Zambia, Zimbabwe) and Southeast Asia (Thailand). Outcomes of interest included adherence to antiretroviral therapy (ART), ART knowledge, viral load data, sexual risk behaviours, sexual risk knowledge, retention in care and linkage to care. Overall, psychosocial interventions for AYPLHIV showed important, small‐to‐moderate effects on adherence to ART (SMD = 0.3907, 95% CI: 0.1059 to 0.6754, 21 studies, n = 2647) and viral load (SMD = −0.2607, 95% CI −04518 to −0.0696, 12 studies, n = 1566). The psychosocial interventions reviewed did not demonstrate significant impacts on retention in care (n = 8), sexual risk behaviours and knowledge (n = 13), viral suppression (n = 4), undetectable viral load (n = 5) or linkage to care (n = 1) among AYPLHIV. No studies measured transition to adult services. Effective interventions employed various approaches, including digital and lay health worker delivery, which hold promise for scaling interventions in the context of COVID‐19.

**Conclusions:**

This review highlights the potential of psychosocial interventions in improving health outcomes in AYPLHIV. However, more research needs to be conducted on interventions that can effectively reduce sexual risk behaviours of AYPLHIV, as well as those that can strengthen engagement in care. Further investment is needed to ensure that these interventions are cost‐effective, sustainable and resilient in the face of resource constraints and global challenges such as the COVID‐19 pandemic.

## 1 INTRODUCTION

Adolescents living with HIV are unlikely to meet the necessary milestones to end the AIDS epidemic by 2030. In 2019, an estimated 1.7 million (1.1 to 2.4 million) adolescents between ages 10 and 19 were living with HIV [1]. Adolescents and young people comprise a growing proportion of new infections globally, and especially in sub‐Saharan Africa, with a decline in mother‐to‐child transmission coupled with persistent HIV incidence [2, 3, 4, 5, 6]. Furthermore, adolescents globally experience gaps in achieving testing, treatment and retention goals, all of which are critical to ending the epidemic [7].

Adolescence and young adulthood is a time characterized by identity exploration, experimentation, risk‐taking and increased vulnerability, during which many lifelong health behaviours become established [8]. For adolescents and young people living with HIV (AYPLHIV), this life stage brings increasing individual responsibilities, for example in managing their own HIV medication, as well as novel and distinct risks to their physical and emotional health. Many AYPLHIV face additional psychosocial challenges during this transitional time, including stigmatization, isolation and fears of rejection [9, 10]. For AYPLHIV who enter romantic and sexual relationships, learning to navigate issues of disclosure and safe sexual practices becomes particularly important [11]. Youth newly diagnosed with HIV, or who have been newly disclosed to, may also face challenges with accepting their status, which can be stressful and result in familial conflict, especially if young people blame their parents for their illness [12]. AYPLHIV have also been found to have poorer outcomes than other age groups in areas such as retention in care [13, 14], treatment adherence [15], HIV knowledge [16] and engagement in care [17]. As the global scale‐up of antiretroviral therapy (ART) has been implemented, AYPLHIV are the only age group with increasing mortality rates [18, 19].

These mortality rates reflect gaps in adolescent HIV care, including low rates of retention in care [7, 17, 20, 21], poor adherence to ART [7, 20, 22, 23, 24], incomplete linkage to care [7, 20, 25, 26] and sexual risk behaviours [27, 28]. In high‐income countries, linkage to and retention in care are lowest among adolescents [20]. Importantly, these care‐related outcomes are related: for example barriers in linking to and being retained in care have downstream effects on adolescents’ ability to adhere to treatment. Barriers to linkage to care may include challenges in accessing health facilities; lack of adolescent‐friendly health services and personnel [29, 30] and inadequate testing and counselling capacity.

In low‐ and middle‐income countries, adolescent disengagement from care presents a major challenge. In these settings, barriers to retention in care include perceived and enacted stigma, fear of disclosure of HIV status to the adolescent or others and poor mental health [20, 31]. Furthermore, studies suggest that adherence to ART may be influenced by age, access to healthcare, intersecting vulnerabilities and co‐morbidities, policies surrounding risk behaviours and mental health [32]. Specifically, younger adolescent age and a history of mental health challenges are significantly associated with poor adherence [33]. The transition between paediatric and adult services has also been highlighted as a particular barrier to retention in care [34]. Current policies are structured towards the needs of adult populations; these must undergo a paradigm shift to comprehensively address the unique needs of adolescent populations [32].

An increased propensity for risk‐taking is a common phenomenon during adolescence [35]; however, for AYPLHIV, these risks may be associated with greater vulnerability due to a number of linked factors [36, 37]. In sub‐Saharan Africa, this group’s vulnerability to high‐risk behaviour may be exacerbated by social and environmental factors including household poverty, orphanhood, gender inequality, stigma and poor accessibility to social or health services [38, 39, 40, 41, 42]. These are the predominant risks studied to date in the quantitative literature; however, a broader set of factors, including intimate partner violence and adolescent sexuality, has emerged from qualitative studies [43]. Moreover, growing evidence suggests that underlying physiological conditions such as HIV‐associated neurocognitive disorders [44], anxiety, and depression [45, 46] increase susceptibility to risk‐taking among AYPLHIV.

Psychosocial interventions are one potential pathway to improve engagement in care and health and behavioural outcomes among AYPLHIV. Psychosocial interventions incorporate psychological, social, and/or behavioural approaches and may include activities, techniques or strategies delivered through interpersonal or information means [47]. These interventions may be broad‐based, targeting multiple outcomes linked to health and wellbeing, or more targeted and directed at specific mechanisms. The implementation of psychosocial interventions may serve as a platform to address underlying health issues for AYPLHIV, and may elicit greater gains than individual treatment options [48, 49]. In addition to targeting specific psychosocial issues, behavioural interventions derived from mental health practice may also contribute to adherence to ART [50]. These types of interventions have been implemented across a range of settings to improve adherence to treatment [51], promote mental health and wellbeing [52, 53] and reduce sexual risk behaviours [54, 55]. However, evidence and experience in applying these interventions to AYPLHIV is needed.

This review investigated the effectiveness of psychosocial interventions for improving engagement in care and health and behavioural outcomes (including clinical HIV outcomes and sexual risk behaviours) for AYPLHIV.

## 2 METHODS

This systematic review and meta‐analysis was developed to inform the 2020 WHO Consolidated Guidelines on the Use of Antiretroviral Drugs for Treating and Preventing HIV Infection, which included a section on service delivery under which this review falls. As part of the WHO guidelines development process, external evidence reviews including systematic reviews and meta‐analyses are commissioned to provide evidence for the group’s deliberations. Our review’s focus was based on the review question set out by the working group coordinating this specific guideline. We followed the standardized systematic review methodology as described in the *WHO Handbook for Guidelines Development, Second Edition* [56]. The systematic review protocol was prospectively registered on PROSPERO (CRD42020197452) and adheres to PRISMA standards of reporting.

### 2.1 Inclusion and exclusion criteria

We included studies published in peer‐reviewed journals between 1 January 2000 and 1 July 2020. Searches were conducted on 1 July 2020; the review itself was completed from 1 July to 1 October 2020. To be eligible, studies had to evaluate psychosocial interventions, defined as interventions using a social, psychological or behavioural approach, or a combination of these two. We aimed to include studies of interventions interpersonal or informational activities, techniques or strategies that targeted behavioural, psychological, interpersonal or social factors with the aim of improving health outcomes [57, 58]. Studies eligible for inclusion could include evaluations conducted as randomized controlled trials, crossover trials, cluster randomized trials or factorial trials. There was no exclusion based on language or geographical region.

We included studies with participants who were male or female adolescents and young people living with HIV/AIDS, between 10 and 24 years of age. When studies had age ranges that extended beyond this limit, they were included if the mean age of the participants was within the specified age range. Interventions could be implemented in an individual, group or combined context. While adolescents and/or young people living with HIV were the main target group, we also included intervention studies with additional participants, such as caregivers. Interventions aimed at improving adolescents’ socioeconomic status, such as through the use of cash transfers, were only included when interventions combined these strategies with a psychosocial component.

The primary comparator for the studies included in this review was cared as usual. In a number of instances, a time‐matched alternative healthcare intervention was used as the control group. Studies comparing two active interventions were not included.

### 2.2 Search methods for identification of publications

Relevant studies were identified by using a set of predetermined search terms to systematically search numerous academic databases, including Cochrane Library, PsycINFO, PubMed and Scopus (see Table S1 for search strategies by the database). A trained information specialist developed and ran the searches, and the search terms were adapted for each database. All identified studies were exported to Covidence [59] where all duplicates were removed. Reviewers, working in pairs, independently reviewed all abstracts. Following the removal of excluded studies, we obtained full‐text versions of relevant studies from academic databases. Two reviewers independently evaluated each paper to determine its eligibility. In cases of disagreement, articles were adjudicated by a senior team member.

### 2.3 Data extraction

We used a standardized data extraction form to organize data extraction from each included study. Two review team members independently extracted key study characteristics, intervention content and delivery, risk of bias and study quality and control group type. Publications that were linked to the same study were merged as one study record. A senior review team member reviewed all discrepancies on a rolling basis.

### 2.4 Types of outcomes

Outcome data were also extracted in duplicate by two independent reviewers, with all discrepancies discussed and changes and corrections to the extracted data recorded. During the extraction process, reviewers identified distinct differences between types of outcomes reported within the domains of sexual risk and adherence to ART. In order to provide a more accurate analysis of the effectiveness of the intervention on a given outcome, sexual risk was divided into two categories for analysis (sexual risk behaviour, sexual risk knowledge) as was adherence (adherence to ART, ART knowledge). For the meta‐analysis, viral load outcomes were divided into three distinct categories to ensure alignment of measures and to capture outcomes as reported by the studies: level of viral load was captured as a continuous variable; viral suppression was captured as a binary variable (<1000 copies/mL), as was undetectable viral load (<200 copies/mL). The final outcomes of interest included: adherence to ART, ART knowledge, retention in care, sexual risk behaviours, sexual risk knowledge, linkage to care, viral load (level), viral suppression, undetectable viral load and improved transitioning to adult services. Outcomes, as reported by study authors, could include self‐report data, biological data (e.g. for viral load), and/or other clinical records. Definitions of outcomes are available in Table S2.

### 2.5 Risk of bias

We used the Cochrane Risk of Bias tool to assess the risk of bias according to the following domains: random sequence generation, allocation concealment, blinding of participants and personnel, blinding of outcome assessment, incomplete outcome data, selective reporting and other sources of bias [60]. For studies that were assessed as cluster randomized controlled trials, four additional areas were assessed: recruitment bias, baseline imbalance, loss of clusters and incorrect analysis.

### 2.6 Data analysis

We classified effect estimates from included studies according to the outcome domain they represented and length of follow‐up. Effects were measured by extracting data from all reported timepoints. Effect estimates were transformed into standardized mean differences (SMD), preferring Cohen's d. Where binary outcomes were reported, we converted odds ratios to Cohen's d using the logit transformation. Each of these transformations were conducted independently by two reviewers.

Meta‐analyses were undertaken using robust variance estimation with random effects in order to account for multiple dependent effect estimates by study. In many cases, one study contributed several effect estimates to one outcome domain. We assumed an intercorrelation of 0.8 within studies. We described heterogeneity in terms of τ² adjusted for clustering and I². Meta‐regression included categorical predictors to describe intervention and population characteristics that may account for heterogeneity in effectiveness. Meta‐regressions were described using the regression coefficient, residual I² and residual τ^2^ The primary analysis compared treatment versus control group. Finally, we used GRADEPro GDT software to determine the certainty of the evidence and followed methodology as described by the GRADE Handbook and the mhGAP guidelines [61, 62].

## 3 RESULTS AND DISCUSSION

**Figure 1:**
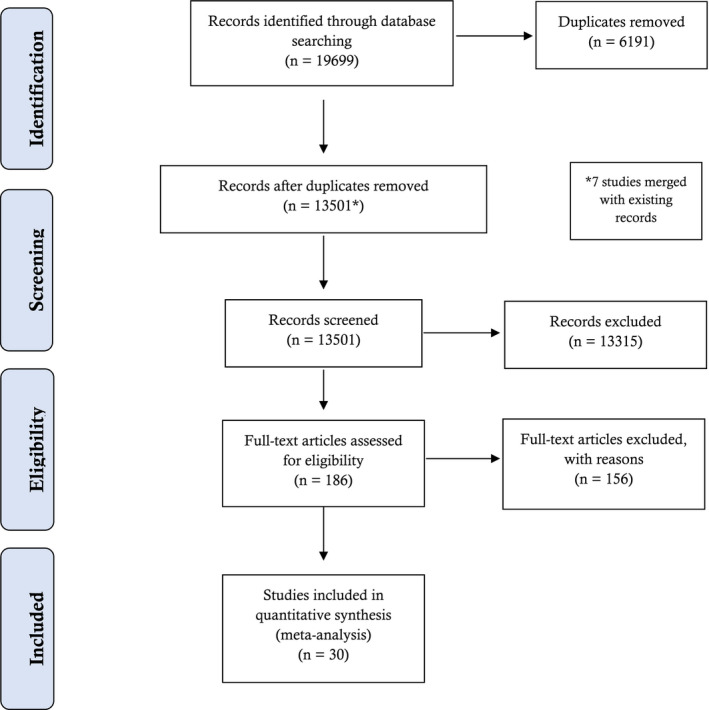
Flow chart of included studies. Reasons for full‐text exclusion: wrong age (n = 80), wrong study design (n = 30), wrong publication type (n = 21), wrong outcomes (n = 19), wrong analysis (n = 3), wrong intervention (n = 1), wrong population (n = 2)

We identified 30 relevant studies for inclusion in this review (Figure 1).

### 3.1 Characteristics of included studies

The majority of studies were conducted in the United States (n = 18, 60%), with the remainder of studies done in Uganda (n = 4) [63, 64, 65, 66, 67], Thailand (n = 3) [68, 69, 70, 71], Zimbabwe (n = 2) [72, 73], Nigeria (n = 1) [74], South Africa (n = 1) [75] and Zambia (n = 1) [76]. Most studies (n = 26, 86.7%) used an individually randomized controlled trial design to evaluate the intervention, whereas fewer (n = 4, 13.3%) used cluster randomization with families or clinic sites as the unit of randomization (Table 1) [63, 67, 71, 72, 77].

**Table 1:** Included studies

Author and year	Country	Study population description and sample	Age (mean)	Female (%)	Programme intent	Outcomes								
						Adherence to ART	ART knowledge	SRH behaviours	SRH knowledge	Retention in care	Linkage to care	Viral load reduction	Viral suppression	Undetectable viral load (<200 copies/mL)
Belzer et al. (2014)/Sayegh et al. (2018)	U.S.	Young people living with HIV (n = 37)	20.43	37.8	To improve adherence and viral control during and following a 24‐week phone‐based intervention.	**X**				**X**		**X**	**X**	**X**
Bermudez et al. (2018)/Ssewamala et al. (2020)	Uganda	Adolescents living with HIV (n = 702)	12.45	56.4	To improve viral suppression through a savings‐led economic empowerment intervention.							**X**		**X**
Berrien et al. (2004)	U.S.	Children and young people living with HIV (n = 37)	10.5	51.4	To improve medication adherence through a home‐based nursing intervention.	**X**							**X**	
Bhana et al. (2014)	South Africa	Pre‐adolescents living with HIV and their caregivers (n = 65)	11.6	51	To improve psychosocial well being through a family‐based, adapted intervention.	**X**								
Bouris et al. (2017)	U.S.	Young Black men who have sex with men and transgender women living with HIV (n = 98)	23.8	0	To improve retention in care, knowledge, and support through a support network intervention.		**X**		**X**					
Brothers et al. (2016)	U.S.	Young women living with HIV (n = 43)	21	100	To reduce onwards transmission, reduce HIV‐related risk behaviour, and empower participants to lead healthy lives			**X**	**X**					
Brown et al. (2016)	U.S.	Young people living with HIV with co‐morbid mental health disorders (n = 42)	21.5	31	To improve adherence and mental health through a manualized, measurement‐guided treatment for depression for adolescents and young adults in care.							**X**		
Chen et al. (2011)/ Naar‐King et al. (2009)	U.S.	Young people living with HIV engaged in risk behaviours (n = 186)	20.5	47.3	To improve motivation, condom use, and viral load, and reduce depression, through a motivational interviewing‐based multi‐risk reduction intervention.	**X**		**X**	**X**			**X**		
Christodoulou et al. (2019)	U.S.	Young people living with HIV (n = 28)	24	7.7	To improve adherence using an adapted active visualization device that demonstrates how ART works in the body.	**X**				**X**		**X**		
Denison et al. (2020)	Zambia	Adolescents and young people living with HIV (n = 273)	19.11	59.3	To improve viral suppression and reduce stigma among through a peer mentor intervention.					**X**			**X**	
Dulli et al. (2020)	Nigeria	Young people living with HIV (n = 349)	21.15	87.7	To promote retention in care, adherence and knowledge through a social media‐based support group intervention.	**X**				**X**				
Garofalo et al. (2016)	U.S.	Poorly adherent young people living with HIV (n = 105)	24.1	18.1	To improve adherence using a two‐way, personalised daily text messaging intervention.	**X**						**X**		**X**
Hosek et al. (2018)	U.S.	Young people newly diagnosed with HIV within the past 12 months (n = 103)	20.17	19.4	To improve engagement in care through an intervention targeting stigma, disclosure, healthy relationships, substance use, and future life planning.	**X**				**X**	**X**			
Jones et al. (2017)	U.S.	Adolescent girls and young women perinatally or behaviourally infected with HIV (n = 34)	22.09	100	To examine the influence of intervention strategies targeting fertility planning, safer conception practices and patient‐provider communication.		**X**	**X**						
Kaihin et al. (2015)	Thailand	Perinatally and behaviourally infected young people living with HIV/AIDS (n = 46)	18.79	56.5	To improve adherence using an empowerment intervention.	**X**								
Lightfoot et al. (2007)	Uganda	Young people living with HIV (n = 100)	18.7	72	To reduce sexual risk behaviours using a culturally adapted version of a previously evaluated efficacious HIV prevention programme.		**X**							
Linnemayr et al. (2017)	Uganda	Young people living with HIV (n = 110)	18.3	63	To improve adherence using SMS reminder messages with and without response options.	**X**								
MacCarthy et al. (2020)	Uganda	Young people living with HIV (n = 155)	NS (range 15‐24)	‐	To improve adherence using a text‐based intervention providing weekly real‐time adherence feedback, based on information from a smart pill box.	**X**								
Mavhu et al. (2020)	Zimbabwe	Adolescents living with HIV (n = 284)	NS (range 13‐19)	52	To improve clinical and social outcomes using a peer‐led differentiated service delivery intervention.	**X**			**X**				**X**	
Mimiaga et al. (2019)	U.S.	Young people living with HIV (n = 60)	19	40	To improve adherence through a targeted problem solving intervention.	**X**								
Naar‐King et al. (2013)	U.S.	Young people with HIV newly prescribed antiretroviral treatment (n = 76)	20.32	19.7	To improve adherence using a brief computer delivered motivational intervention.	**X**						**X**		**X**
Naar‐King et al. (2006)	U.S.	Young people living with HIV (n = 51)	21	49	To target multiple health risk behaviours using a brief individual motivational intervention.			**X**				**X**		
Naar‐King et al. (2008)	U.S.	Young people living with HIV (n = 65)	21.09	47.7	To reduce risk behaviours and viral load using a Motivational Enhancement Therapy (MET) intervention.			**X**				**X**		
Nestadt et al. (2019)	Thailand	Young adolescents with perinatal HIV transmission and their caregivers (n = 88)	12.3	49	To improve adherence and reduce psychosocial challenges through an adapted intervention.	**X**	**X**							
Rongkavilit et al. (2013, 2014)	Thailand	Young people living with HIV, subset examining young HIV positive men who have sex with men (n = 110)	22.5	18.5	To reduce sexual risk behaviours through a motivational interviewing‐based intervention.	**X**		**X**	**X**			**X**		
Rotheram‐Borus et al. (2004)	U.S.	Young people living with HIV engaged in risk behaviours (n = 175)	23 (median)	22	To reduce risky sexual behaviours and improve health practices using a preventive intervention.	**X**		**X**						
Spratt et al. (2017)	U.S.	Nonadherent young people living with HIV (n = 12)	17.1	75	To improve adherence through an intervention using an electronic pillbox and cell phone texting with personalised motivational interviewing strategies.	**X**								
Webb et al. (2018)	U.S.	Young people living with HIV (n = 72)	18.71	47.2	To improve psychological symptoms and HIV management through mindfulness‐based stress reduction (MBSR).	**X**								**X**
Whiteley et al. (2018)	U.S.	Young people living with HIV (n = 61)	22.4	21.3	To improve adherence and knowledge and reduce viral load using a game‐based digital intervention.	**X**	**X**					**X**		
Willis et al. (2019)	Zimbabwe	Adolescents living with HIV (n = 100)	NS (range 10 to 15)	61.7	To improve linkage to services, retention in care, adherence and psychosocial wellbeing through a peer‐delivered intervention.	**X**				**X**	**X**			

The majority of studies recruited both male and female participants, with one study including only male participants [78] and two studies recruiting only female participants [79, 80]. The total sample size per study ranged from n = 12 to n = 702 participants (mean = 135.17, SD = 154.7), and a total of n = 4055 participants were included in the review.

Participants’ mean age, reported across 26 of the 30 studies, was 19.33 years (SD = 3.76). Other studies provided age ranges or median age. Most studies targeted individuals who were 15 years and older (n = 22, 73.3%), whereas others targeted younger participants (n = 4, 13.3%) [63, 67, 68, 73, 75] or a broader range of ages.

### 3.2 Intervention implementation

#### 3.2.1 Settings

Clinic‐based interventions comprised half of the studies (n = 15). A further six interventions were delivered in a clinical setting combined with another location or strategy (e.g. digital) [64, 72, 73, 74, 81, 82]. Other in‐person interventions utilized home‐based (n = 1) [83], university‐based (n = 1) [80] and community‐centre settings (n = 1) [78]. Four studies took place completely digitally [65, 66, 84, 85], and one study was implemented telephonically [86, 87]. One study used a combination of these settings and strategies to deliver their intervention.

#### 3.2.2 Personnel

Health providers were used to deliver the interventions in one‐third of studies (n = 10), including three by nurses [64, 71, 83], one by a social worker [78] and others by clinicians or mental health professionals [69, 77, 88, 89, 90, 91, 92]. Four studies involved peer implementers, in some cases relying on professional support to implement interventions [72, 73, 76, 93]. Five interventions were delivered by lay counsellors or facilitators [74, 79, 86, 87, 94, 95]. Additionally, five interventions had no delivery personnel involved and were delivered by digital means only (one of these was delivered in a hybrid clinical‐digital setting as described above) [65, 66, 82, 84, 85]. Four interventions were delivered using a combination of personnel, and two studies did not specify the intervention implementers.

#### 3.2.3 Delivery format

Over a third of studies included interventions that had a remote delivery component (n = 11, digital, SMS and telephonic calls). Sixteen studies specified total contact time, which ranged from 30 minutes to 36 hours.

#### 3.2.4 Target group

All included studies were targeted toward AYPLHIV. Certain studies targeted specific groups, including: AYPLHIV struggling with treatment adherence (n = 3, 10%) [71, 81, 84], those newly diagnosed with HIV (n = 2, 6.7%) [82, 93], those engaged in risky behaviours (n = 2, 6.7%) [88, 89, 96], those diagnosed with a mental disorder (n = 1, 3.3%) [77] and young Black men who have sex with men and transgender women (n = 1, 3.3%) [78].

#### 3.2.5 Adolescent engagement and parental involvement

AYPLHIV participated in discussions around the development, design and/or implementation of the intervention—or participated in actually implementing the intervention as peer facilitators—in over half of studies (n = 16, 53.3%). Six interventions were designed to include parental or caregiver participation, engaging them along with their children, or in less direct engagement (20.0%) [63, 67, 68, 73, 75, 76, 83].

### 3.3 Intervention type and theoretical framework of interventions

Interventions were broadly categorized into interpersonal or informational [97]. Interpersonal interventions included counselling, motivational interviewing or cognitive‐behavioural therapeutic techniques. Informational interventions, instead, employed predominantly one‐way information delivery and/or messaging. Within our sample, 86.7% of studies (n = 26) involved interpersonal interventions, with the remainder utilizing informational interventions [65, 66, 84, 95]. Of the 30 included studies, most interventions (n = 25, 83.3%) were based on a designated theoretical approach or orientation. A smaller number (n = 5, 16.7%) of studies evaluated interventions that could be classified as non‐specific, as they utilized strategies that were more generic to psychosocial interventions [63, 65, 67, 72, 73, 76]. Interventions included those based on principles of motivational interviewing (n = 7, 23.3%) [69, 70, 81, 88, 89, 91, 92, 96, 98], cognitive‐behavioural therapeutic techniques (n = 2, 6.7%) [64, 77] and social action theory (n = 2, 6.7%) [68, 75]. Other studies used theoretical principles such as economic empowerment, peer support, mindfulness and behavioural economics. Additionally, some studies used a combination of theoretical principles.

### 3.4 Risk of bias

Overall, studies were assessed as having a low risk of bias in domains of selective reporting and other bias (see Figure S1). Most studies were assessed as having an unclear risk of bias for random sequence generation (n = 18, 60.0%) and allocation concealment (n = 23, 76.7%) due to poor reporting. Over one‐quarter of studies were assessed as having a high risk of bias for performance bias (n = 8, 26.7%); the majority of the remaining studies were at unclear risk (n = 18, 60.0%), predominantly due to poor reporting combined with a strong likelihood of unblinding. Only five studies were at high risk for detection bias, mostly due to self‐reported outcomes by unblinded participants, but half of the studies (n = 15) were at unclear risk of detection bias. Nearly a quarter of studies reported high attrition, with insufficient information or inappropriate approaches to handling missing data, leading to a high risk of bias judgments (n = 7, 76.7%), whereas only four studies in the attrition domain were judged as unclear (13.3%). For cluster‐randomized trials, the additional risk of bias criteria was applied; baseline imbalance and loss of clusters presented a low risk of bias, whereas high risk of bias for recruitment bias and incorrect analysis techniques were identified in two studies each.

### 3.5 Meta‐analysis results

Psychosocial interventions for AYPLHIV showed important, small‐to‐moderate effects on adherence to ART (SMD = 0.3907, 95% CI: 0.1059 to 0.6754, n = 2647, very low certainty evidence) and level of viral load (SMD = −0.2607, 95% CI −04518 to −0.0696, n = 1566, very low certainty evidence). For adherence to ART, substantial significant heterogeneity was identified with an I² of 80%. For viral load, heterogeneity was below the pre‐specified threshold, at 60%. Retention in care (very low certainty evidence) showed non‐significant results, as did sexual risk behaviours (very low certainty evidence) and sexual risk knowledge (low certainty evidence). The confidence intervals for viral suppression and undetectable viral load findings, dichotomized and converted to odds ratios, were wide and spanned unimportant benefit as well as large, important benefit; this made these outcomes less conclusive than viral load measured as a continuous variable. All three of these outcomes had very low certainty evidence. Finally, ART knowledge was statistically significant with high certainty of evidence, but its effect size was not clinically relevant (SMD = 0.1263, 95% CI 0.1131 to 0.1395). Linkage to care and improved transitioning to adult services were not included in the meta‐analysis due to insufficient data. Table 2 shows these findings. Forest plots for all outcomes are available in Table S3.

**Table 2:** Meta‐analysis of all outcomes at all time points

Outcomes (number of studies)	Effect size (SMD)	*p*‐value	95% confidence intervals		I² (%)
Adherence to ART (n = 21)	0.3907	0.0098*	0.1059	0.6754	80
ART knowledge (n = 2)	*0.1263*	*0.0052*	*0.1131*	*0.1395*	*0*
Retention in care (n = 8)	0.2823	0.1630	−0.1425	0.7072	88
Sexual risk behaviours (n = 9)	0.3261	0.1534	−0.1542	0.8064	82
Sexual risk knowledge (n = 4)	*0.2671*	*0.0899*	*−0.0957*	*0.6298*	*45*
Linkage to care (n = 1, not meta‐analysed)	–	–	–	–	
Viral load (n = 12)	−0.2607	0.0157*	−0.4518	−0.0696	60
Viral suppression (OR) (n = 4)	1.938		1.001	3.756	34
Undetectable viral load (OR) (n = 5)	1.827		1.074	3.110	34
Improved transitioning to adult services	–	–	–	–	

Models in italics are indicative only, given the statistical estimation procedures used. For viral load, a negative effect size denotes a beneficial effect. For all other outcomes, a positive effect size denotes a beneficial effect.

* *p* < 0.05.

## 4 DISCUSSION

In this review, we synthesized and meta‐analysed randomized controlled trials assessing the effect of psychosocial interventions on engagement in care and health and behavioural outcomes for AYPLHIV. While a growing body of research has attempted to understand drivers of adolescent engagement in HIV care [99, 100, 101], as well as effective modes of improving health outcomes and reducing sexual risk behaviours in this age group [102, 103], our systematic review and meta‐analysis represents an important step toward establishing evidence for the effectiveness of psychosocial interventions to improve these outcomes for AYPLHIV.

Our findings showed that intervention participants demonstrated better adherence to ART and reductions in viral load among intervention participants when compared to controls. These types of interventions may be able to encourage positive behavioural and psychosocial changes in AYPLHIV, especially in regard to HIV treatment outcomes. The psychosocial interventions that we identified tended to rely on evidence‐based approaches that have also been shown to be effective at improving adherence among adults living with HIV, including community‐based adherence support and digital platforms [104]. These changes may also stem from high levels of support and investment from study team members, enabling adolescents living in resource‐limited communities to engage more fully with the intervention than they would have been able to do within the standard of care, as well as from reporting bias, specifically in self‐reported measures [105].

While psychosocial interventions showed promise in improving HIV‐specific outcomes, other outcomes related to engagement in care and behavioural outcomes were not found to be affected by these interventions. These types of outcomes, however, may be important to consider when aiming for longer‐term, sustained improvements in health and health service engagement, and should be carefully considered in the design and implementation of this type of intervention. It is possible, for instance that these outcomes may require bolstering by multiple, complementary approaches. Retention in care is one example, where AYPLHIV may require a broader set of supports alongside individual skills to improve and sustain engagement in care. Some interventions included members of the participant’s social network, including family members, to support retention in care [73, 106, 107]. Longer follow‐up periods may also enable more accurate measures of retention in care, whereas many of these evaluations only followed participants for a short period following the end of the intervention.

Our meta‐analysis showed limited effectiveness for improving outcomes related to sexual risk behaviours and knowledge. This is an important finding, especially given the intertwined risks of early, unintended pregnancy and secondary HIV transmission, especially in sub‐Saharan Africa [108, 109]. Sexual risk behaviours in AYPLHIV have been found to be driven by a combination of individual, relationship, family, community and structural factors [110, 111]. Although a number of psychosocial interventions delivered content focused on influencing decision making about sexual risk behaviours and sexual negotiation skills [79], the overall aim of these studies was not linked specifically to sexual health or mitigating associated risk behaviours. It may be that adolescents require more specific skills related to sexual risk and sexual and reproductive health, such as information and/or education about condom use and contraception, as well as supports such as caregiver engagement to improve communication, which has been found to work in more general adolescent populations [112]. For AYPLHIV, psychosocial interventions may require more targeted attention to sexual risk, in lieu of more broad‐based theoretical approaches such as motivational interviewing and goal‐setting.

Some outcomes were rarely or not reported, and our findings point to the need for additional research areas with populations of AYPLHIV. Only one study investigated linkage to care, and no studies measured improved transitioning to adult services. Linkage to care is important along the entire HIV continuum of care, from prevention of mother‐to‐child transmission along to adult‐focused testing, counselling and treatment [113, 114, 115, 116], and should be included as an outcome of interest for psychosocial interventions in studies with all ages. Interventions to improve transitioning to adult services are critically important for AYPLHIV and must be examined to leverage the potential role of psychosocial support in maintaining service engagement for young people.

### 4.1 Implications for research

This review elicits two critical, intertwined considerations for implementing interventions for AYPLHIV where they are most needed—adapting psychosocial interventions for both demographic/age group and geographical setting. The first consideration relates to the adaptation of interventions developed for adult populations. A substantial number of the studies detailed consultative processes with adolescents and young people, such as youth advisory groups [82], preliminary interviews with AYPLHIV [107, 117, 118], focus groups and workshops [74, 119, 120, 121], youth‐designed personalized content [122] and involvement of peer mentors [72, 73, 78, 81, 123]. These processes are vital to ensure that interventions are implemented with full consideration of what works best for younger HIV‐affected populations [124]. Notably, some sub‐groups of AYPLHIV were not specifically targeted by the studies we found, including pregnant adolescents and young women, young people living with disabilities and young people engaged in sex work and transactional sex.

Secondly, while interventions were distributed across a number of countries—including sub‐Saharan Africa, where the burden of HIV among young people is greatest—our review showed that these interventions are still most commonly implemented in the United States. Our final study subset reflected nearly double the number of studies in the United States as compared to all other countries, although these studies often engaged high‐adversity sub‐populations. More intervention efficacy and effectiveness research with AYPLHIV should be conducted in the regions with the highest burden of HIV, in particular in Eastern and Southern Africa, as well as in other countries with more limited resources and specific HIV‐affected populations of young people [100, 111]. In low‐resource, high‐burden settings in particular, cost‐effectiveness research and other forms of cost‐related data are needed to ensure policy buy‐in, and to build evidence on what works for this growing population [72]. Specifically, investigating costs related to integrating psychosocial interventions with existing HIV services is much‐needed, in order to establish evidence that might add fewer additional costs but contribute to greater downstream effects on the wellbeing of AYPLHIV. While implementing interventions adapted from high‐income settings may be a more feasible way to translate the best evidence to new settings, it is also imperative that adaptations allow for locally appropriate contextual input, to ensure that core content can be effectively delivered and received [125, 126, 127]. Moreover, considering the stark socioeconomic inequities many AYPLHIV may confront, it is important to note that psychosocial interventions alone may not adequately address the challenges this population faces. It is likely that a combination of different kinds of support at different levels may be required to meet the needs of AYPLHIV [128, 129]. Researchers developing and testing interventions in the near future could also consider adopting family‐ and peer‐based intervention models, as well as relationship safety interventions, to provide additional structural supports.

Overall, given the very low certainty of evidence we found, more high‐quality research in this area is needed. This is particularly important considering the significant burden of poor HIV‐linked outcomes in this population. Furthermore, to facilitate comparability of findings across settings, future research should use standard definitions.

### 4.2 Implications for policy and practice

Our review also elicits a set of implications for policy and practice related to HIV programming. Lay health counsellors with specific HIV‐focused training have been leveraged to positive effect among adult populations in resource‐constrained settings [74], and may be a feasible cadre to deliver support to younger populations living with HIV[72, 73]. Where lay workers have been deployed with adult populations, they are often trained in counselling skills [50], and may be employed as part of government health systems. They also tend to come from the general communities in which interventions are implemented [130]. Addressing some of the broader challenges facing AYPLHIV through focusing on strengthening interpersonal skills for implementers of all types may be one way to deliver more holistic, responsive interventions at low cost.

In order to improve the health outcomes of AYPLHIV during their transition to adulthood, and equip them with skills and knowledge to manage their own health and wellbeing, psychosocial interventions need to respond to adolescents’ specific needs. Many of the studies included in this review were attuned to this need, and involved AYPLHIV at various stages of the design, refinement, implementation and evaluation of these interventions. This approach should be emphasized in the process of introducing and implementing psychosocial interventions with adolescent and young populations, who are likely to respond more favourably to interventions that are co‐designed and adapted [131]. In programming as well as in research, representation of more at‐risk sub‐groups of AYPLHIV is also necessary.

Importantly, in the context of the COVID‐19 pandemic, new approaches to reaching AYPLHIV may be required. This population is facing marked disruptions in accessing healthcare, and also encountering additional challenges related to the social and economic impact of COVID‐19 and periods of extended lockdowns [132, 133]. While these challenges have been explored in the immediate emergency response, it is also critical to consider the pandemic’s longer term impact on how vulnerable populations of adolescents are reached and retained in supportive interventions [134]. Our review presents evidence that psychosocial interventions can benefit adherence and viral load outcomes, but might be bolstered by additional considerations around mechanisms by which they can retain their effectiveness and be integrated into other settings that are COVID‐responsive. Digitally based interventions, which were well‐represented among our sample in both high‐ and low‐income settings, are one avenue for expanding the reach of interventions while managing the challenges posed by COVID‐19, including the need for physical distancing and limited travel [135]. Digital interventions may have other benefits such as mitigating fear of unintended disclosure or stigma among AYPLHIV, and may also present attractive and more innovative formats for engaging youth [135, 136]. Similarly, digital interventions are increasingly accessible through mobile phones, enabling rapid and effective reach while upholding safety precautions. It is worth noting that data, airtime and electricity for charging phones have a cost, and COVID‐19 has placed many families in dire financial circumstances where these means may be less accessible than before. It may also be important to consider how to strengthen sexual risk elements of psychosocial interventions, especially within digitally based interventions, for those adolescents and young people who are unable to easily access clinical services, or who may benefit more from alternative approaches to education and care [136, 137, 138].

Although we did not place a language restriction on our search, the final studies examined were all in English. The studies we identified had fewer behavioural outcomes (such as sexual and reproductive health) than HIV‐specific outcomes, and more research with such outcomes is required. Additionally, our findings should be considered in the context of the included studies, which do not accurately represent the geographic distribution of HIV infection among adolescents and young people globally. Despite these limitations, we feel that the studies included in this review reflect a range of diverse psychosocial interventions and ages, making it a valuable addition to the expanding evidence about adolescents living with HIV.

## 5 CONCLUSIONS

This review found that psychosocial interventions for AYPLHIV resulted in small‐to‐moderate beneficial effects on adherence to ART and viral load. More research is needed to understand the long‐term impacts of these types of interventions, especially in light of persistent rates of HIV infection in this age group. Interventions designed with and for adolescents and young people, and implemented in the highest burden regions, are also needed to increase the accuracy and relevance of the evidence base. To this end, cost‐effective solutions, and research accompanying them, are urgently needed. Finally, it is critical to assess the emergent consequences of the COVID‐19 pandemic on how AYPLHIV can access and engage in care and achieve optimal health and wellbeing while navigating the transition to adulthood.

## Competing interests

The authors have declared no conflict of interest.

## Authors’ contributions

CAL conceptualized and drafted the manuscript. SS led the review research team, and CAL, SDT, TK, YC, NA, MB and DTP contributed as members of the core research team to running searches, screening articles, extracting data and preparing data for analysis, as well as contributing to drafting specific sections of the manuscript. AB conducted all risk of bias assessments and led the GRADE process. GJMT conducted all statistical analyses. WA, NF, MP and MV were primarily responsible for defining the review question and contributing to the guidelines development protocol and final guidelines process. DM, NW, NASA and AA contributed to the protocol, discussion and provision of supporting resources. All authors contributed to reviewing, finalizing and approving the final manuscript.

## Abbreviations

ART, antiretroviral therapy; AYP, adolescents and young people; AYPLHIV, adolescents and young people living with HIV; CI, confidence interval; HIV, human immunodeficiency virus; SMD, standardized mean difference.

## Supporting information


**Table S1**. Search strategy

**Table S2**. List of definitions and examples of operationalized terms

**Table S3**. Forest plots for all outcomes

**Figure S1**. Risk of bias diagram

## References

[1] UNAIDS . Regional AIDS Info. 2019. Available from: http://aidsinfo.unaids.org

[2] Shapiro RL , Hughes MD , Ogwu A , Kitch D , Lockman S , Moffat C , et al. Antiretroviral regimens in pregnancy and breast‐feeding in Botswana. N Engl J Med. 2010;362(24):2282–94. 20554983 10.1056/NEJMoa0907736 PMC2999916

[3] World Health Organization . Antiretroviral drugs for treating pregnant women and preventing HIV infection in infants: recommendations for a public health approach‐2010 version. 2010: World Health Organization . 26180894

[4] Lolekha R , Boonsuk S , Plipat T , Martin M , Tonputsa C , Punsuwan N , et al. Elimination of mother‐to‐child transmission of HIV—Thailand. Morb Mortal Wkly Rep. 2016;65(22):562–6. 10.15585/mmwr.mm6522a2 27281244

[5] Kouanda S , Tougri H , Cissé M , Simporé J , Pietra V , Doulougou B , et al. Impact of maternal HAART on the prevention of mother‐to‐child transmission of HIV: results of an 18‐month follow‐up study in Ouagadougou, Burkina Faso. AIDS care. 2010;22(7):843–50. 20635248 10.1080/09540120903499204

[6] Wong VJ , Murray KR , Phelps BR , Vermund SH , McCarraher DR . Adolescents, young people, and the 90–90–90 goals: a call to improve HIV testing and linkage to treatment. AIDS (London, England). 2017;31 Suppl 3:S191. 10.1097/QAD.0000000000001539 PMC5497776 28665876

[7] Wood SM , Dowshen N , Lowenthal E . Time to improve the global human immunodeficiency virus/aids care continuum for adolescents: a generation at stake. JAMA Pediatrics. 2015;169(7):619–20. 25985061 10.1001/jamapediatrics.2015.58 PMC4494868

[8] Barry MM , Clarke AM , Jenkins R , Patel V . A systematic review of the effectiveness of mental health promotion interventions for young people in low and middle income countries. BMC Public Health. 2013;13(1):835. 24025155 10.1186/1471-2458-13-835 PMC3848687

[9] World Health Organization . Psychosocial Wellbeing. 2019. [cited 2020 Oct 1]. Available from: http://apps.who.int/adolescent/hiv‐testing‐treatment/page/Psychosocial_well_being

[10] Vranda MN , Mothi SN . Psychosocial issues of children infected with HIV/AIDS. Indian J Psychol Med. 2013;35(1):19. 23833337 10.4103/0253-7176.112195 PMC3701354

[11] Friedman Nestadt D , Lakhonpon S , Pardo G , Saisaengjan C , Gopalan P , Bunupuradah T , et al. A qualitative exploration of psychosocial challenges of perinatally HIV‐infected adolescents and families in Bangkok, Thailand. Vulnerable Child Youth Stud. 2018;13(2):158–69. 30344614 10.1080/17450128.2017.1356947 PMC6190906

[12] Murphy DA . HIV‐positive mothers' disclosure of their serostatus to their young children: a review. Clin Child Psychol Psychiatry. 2008;13(1):105–22. 18411869 10.1177/1359104507087464 PMC2384146

[13] Evans D , Menezes C , Mahomed K , Macdonald P , Untiedt S , Levin L , et al. Treatment outcomes of HIV‐infected adolescents attending public‐sector HIV clinics across Gauteng and Mpumalanga, South Africa. AIDS Res Human Retroviruses. 2013;29(6):892–900. 23373540 10.1089/aid.2012.0215 PMC3653371

[14] Auld AF , Agolory SG , Shiraishi RW , Wabwire‐Mangen F , Kwesigabo G , Mulenga M , et al. Antiretroviral therapy enrollment characteristics and outcomes among HIV‐infected adolescents and young adults compared with older adults—seven African countries, 2004–2013. MMWR Morb Mortal Wkly Rep. 2014;63(47):1097. 25426651 PMC5779521

[15] Maskew M , Brennan AT , MacPhail AP , Sanne IM , Fox MP . Poorer ART outcomes with increasing age at a large public sector HIV clinic in Johannesburg, South Africa. J Int Assoc Phys AIDS Care. 2012;11(1):57–65. 10.1177/1545109711421641 PMC3272319 21951728

[16] Idele P , Gillespie A , Porth T , Suzuki C , Mahy M , Kasedde S , et al. Epidemiology of HIV and AIDS among adolescents: current status, inequities, and data gaps. JAIDS J Acquir Immune Defic Syndr. 2014;66:S144–53. 24918590 10.1097/QAI.0000000000000176

[17] Zanoni BC , Mayer KH . The adolescent and young adult HIV cascade of care in the United States: exaggerated health disparities. AIDS Patient Care STDs. 2014;28(3):128–35. 24601734 10.1089/apc.2013.0345 PMC3948479

[18] Slogrove AL , Mahy M , Armstrong A , Davies MA . Living and dying to be counted: What we know about the epidemiology of the global adolescent HIV epidemic. J Int AIDS Soc. 2017;20:21520. 28530036 10.7448/IAS.20.4.21520 PMC5719718

[19] UNICEF . For Every Child, End AIDS: Seventh Stocktaking Report, UNICEF, Editor. New York, NY: UNICEF; 2016.

[20] Enane LA , Vreeman RC , Foster C . Retention and adherence: global challenges for the long‐term care of adolescents and young adults living with HIV. Curr Opin HIV AIDS. 2018;13(3):212–9. 29570471 10.1097/COH.0000000000000459

[21] Lamb MR , Fayorsey R , Nuwagaba‐Biribonwoha H , Viola V , Mutabazi V , Alwar T , et al. High attrition before and after ART initiation among youth (15–24 years of age) enrolled in HIV care. AIDS (London, England). 2014;28(4):559. 10.1097/QAD.0000000000000054 PMC4517438 24076661

[22] Fauci AS , Folkers GK . Toward an AIDS‐free generation. JAMA. 2012;308(4):343–4. 22820783 10.1001/jama.2012.8142

[23] Lowenthal ED , Bakeera‐Kitaka S , Marukutira T , Chapman J , Goldrath K , Ferrand RA . Perinatally acquired HIV infection in adolescents from sub‐Saharan Africa: a review of emerging challenges. Lancet Infect Dis. 2014;14(7):627–39. 24406145 10.1016/S1473-3099(13)70363-3 PMC4074242

[24] Gardner EM , McLees MP , Steiner JF , del Rio C , Burman WJ . The spectrum of engagement in HIV care and its relevance to test‐and‐treat strategies for prevention of HIV infection. Clin Infect Dis. 2011;52(6):793–800. 21367734 10.1093/cid/ciq243 PMC3106261

[25] Kahana SY , Mahy M , Armstrong A , Davies MA . Rates and correlates of antiretroviral therapy use and virologic suppression among perinatally and behaviorally infected HIV+ youth linked to care in the United States. J Acquir Immune Defic Syndr. 2015;68(2):169. 25590270 10.1097/QAI.0000000000000408 PMC4312477

[26] MacPherson P , MacPherson EE , Mwale D , Squire SB , Makombe SD , Corbett EL , et al. Barriers and facilitators to linkage to ART in primary care: a qualitative study of patients and providers in Blantyre, Malawi. J Int AIDS Soc. 2012;15:18020. 23336700 10.7448/IAS.15.2.18020 PMC3535694

[27] Bekker LG , Johnson L , Wallace M , Hosek S . Building our youth for the future. J Int AIDS Soc. 2015;18:20027. 25724512 10.7448/IAS.18.2.20027 PMC4344540

[28] Sandy PT , Vhembo T , Molotsi TK . Sexual behaviour among adolescents living with the human immunodeficiency virus in Zimbabwe: educational implications. Afr J AIDS Res. 2019;18(2):130–7. 31282303 10.2989/16085906.2019.1621910

[29] Philbin MM , Tanner AE , DuVal A , Ellen J , Kapogiannis B , Fortenberry JD . Linking HIV‐positive adolescents to care in 15 different clinics across the United States: creating solutions to address structural barriers for linkage to care. AIDS Care. 2014;26(1):12–9. 23777542 10.1080/09540121.2013.808730 PMC3872213

[30] Kempf M‐C , McLeod J , Boehme AK , Walcott MW , Wright L , Seal P , et al. A qualitative study of the barriers and facilitators to retention‐in‐care among HIV‐positive women in the rural southeastern United States: implications for targeted interventions. AIDS Patient Care STDs. 2010;24(8):515–20. 20672971 10.1089/apc.2010.0065

[31] Okonji EF , Mukumbang FC , Orth Z , Vickerman‐Delport SA , Van Wyk B . Psychosocial support interventions for improved adherence and retention in ART care for adolescents and young people living with HIV: a scoping review. BMC Public Health. 2020;20(1):1. 33261566 10.1186/s12889-020-09717-y PMC7708133

[32] Lall P , Lim SH , Khairuddin N , Kamarulzaman A . An urgent need for research on factors impacting adherence to and retention in care among HIV‐positive youth and adolescents from key populations. J Int AIDS Soc. 2015;18:19393. 25724503 10.7448/IAS.18.2.19393 PMC4344535

[33] Murphy DA , Belzer M , Durako SJ , Sarr M , Wilson CM , Muenz LR . Longitudinal antiretroviral adherence among adolescents infected with human immunodeficiency virus. Arch Pediatr Adolesc Med. 2005;159(8):764–70. 16061785 10.1001/archpedi.159.8.764

[34] Foster C , Fidler S . Optimizing HIV transition services for young adults. Curr Opin Infect Dis. 2018;31(1):33–8. 29210712 10.1097/QCO.0000000000000424

[35] Steinberg L . A social neuroscience perspective on adolescent risk‐taking. Develop Rev. 2008;28(1):78–106. 10.1016/j.dr.2007.08.002 PMC2396566 18509515

[36] Bakeera‐Kitaka S , Nabukeera‐Barungi N , Nöstlinger C , Addy K , Colebunders R . Sexual risk reduction needs of adolescents living with HIV in a clinical care setting. AIDS Care. 2008;20(4):426–33. 18449819 10.1080/09540120701867099

[37] Nöstlinger C , Bartoli G , Gordillo V , Roberfroid D , Colebunders R . Children and adolescents living with HIV positive parents: emotional and behavioural problems. Vulner Child Youth Stud. 2006;1(1):29–43.

[38] Birdthistle IJ , Floyd S , Machingura A , Mudziwapasi N , Gregson S , Glynn JR . From affected to infected? Orphanhood and HIV risk among female adolescents in urban Zimbabwe. AIDS. 2008;22(6):759–66. 18356606 10.1097/QAD.0b013e3282f4cac7

[39] Swendeman D , Rotheram‐Borus MJ , Comulada S , Weiss R , Ramos ME . Predictors of HIV‐related stigma among young people living with HIV. Health Psychology. 2006;25(4):501. 16846325 10.1037/0278-6133.25.4.501 PMC2392891

[40] Clark S . Early marriage and HIV risks in sub‐Saharan Africa. Stud Family Plan. 2004;35(3):149–60. 10.1111/j.1728-4465.2004.00019.x 15511059

[41] Nakigozi G , Atuyambe L , Kamya M , Makumbi FE , Chang LW , Nakyanjo N , et al. A qualitative study of barriers to enrollment into free HIV care: perspectives of never‐in‐care HIV‐positive patients and providers in Rakai, Uganda. BioMed Res Int. 2013;2013:1–7. 10.1155/2013/470245 PMC3766571 24058908

[42] Foster G , Williamson J . A review of current literature on the impact of HIV/AIDS on children in sub‐Saharan Africa. AIDS. 2000;14:S275–84. 11086871

[43] Mackworth‐Young CRS , Bond V , Wringe A , Konayuma K , Clay S , Chiiya C , et al. “My mother told me that I should not”: a qualitative study exploring the restrictions placed on adolescent girls living with HIV in Zambia. J Int AIDS Soc. 2017;20:e25035. 10.1002/jia2.25035 PMC5810345 29219248

[44] Anand P , Springer SA , Copenhaver MM , Altice FL . Neurocognitive impairment and HIV risk factors: a reciprocal relationship. AIDS Behav. 2010;14(6):1213–26. 20232242 10.1007/s10461-010-9684-1 PMC2906682

[45] Murphy DA , Durako SJ , Moscicki A‐B , Vermund SH , Ma Y , Schwarz DF , et al. No change in health risk behaviors over time among HIV infected adolescents in care: role of psychological distress. J Adolesc Health. 2001;29(3):57–63. 11530304 10.1016/s1054-139x(01)00287-7

[46] Ssewanyana D , Mwangala PN , Van Baar A , Newton CR , Abubakar A . Health risk behaviour among adolescents living with HIV in sub‐Saharan Africa: a systematic review and meta‐analysis. BioMed Res Int. 2018;2018:1–18. 10.1155/2018/7375831 PMC5896333 29789804

[47] Gonzalez ML , Butler AS , England MJ . Psychosocial interventions for mental and substance use disorders: a framework for establishing evidence‐based standards. Washington, DC: National Academies Press; 2015. 26203478

[48] Himelhoch S , Medoff DR , Oyeniyi G . Efficacy of group psychotherapy to reduce depressive symptoms among HIV‐infected individuals: a systematic review and meta‐analysis. AIDS Patient Care STDS. 2007;21(10):732–9. 17949272 10.1089/apc.2007.0012

[49] Kuhns LM , Hotton AL , Garofalo R , Muldoon AL , Jaffe K , Bouris A , et al. An Index of multiple psychosocial, syndemic conditions is associated with antiretroviral medication adherence among HIV‐positive youth. AIDS Patient Care STDS. 2016;30(4):185–92. 27028184 10.1089/apc.2015.0328 PMC4827312

[50] Freeman MC , Patel V , Collins PY , Bertolote JM . Integrating mental health in global initiatives for HIV/AIDS. Br J Psychiatry. 2005;187(1):1–3. 15994563 10.1192/bjp.187.1.1

[51] Spaan P , van Luenen S , Garnefski N , Kraaij V . Psychosocial interventions enhance HIV medication adherence: a systematic review and meta‐analysis. J Health Psychol. 2020;25(10–11):1326–40. 29417851 10.1177/1359105318755545 PMC7480021

[52] van Luenen S , Garnefski N , Spinhoven P , Spaan P , Dusseldorp E , Kraaij V . The benefits of psychosocial interventions for mental health in people living with HIV: a systematic review and meta‐analysis. AIDS Behav. 2018;22(1):9–42. 28361453 10.1007/s10461-017-1757-y PMC5758656

[53] Sherr L , Clucas C , Harding R , Sibley E , Catalan J . HIV and depression–a systematic review of interventions. Psychol Health Med. 2011;16(5):493–527. 21809936 10.1080/13548506.2011.579990

[54] Meader N , Semaan S , Halton M , Bhatti H , Chan M , Llewellyn A , et al. An international systematic review and meta‐analysis of multisession psychosocial interventions compared with educational or minimal interventions on the HIV sex risk behaviors of people who use drugs. AIDS Behav. 2013;17(6):1963–78. 23386132 10.1007/s10461-012-0403-y

[55] van Empelen P , Kok G , van Kesteren NMC , van den Borne B , Bos AER , Schaalma HP . Effective methods to change sex‐risk among drug users: a review of psychosocial interventions. Soc Sci Med. 2003;57(9):1593–608. 12948569 10.1016/s0277-9536(02)00557-9

[56] World Health Organization . WHO handbook for guideline development, 2nd ed. Geneva: World Health Organization; 2014.

[57] Richter L , Foster G , Sherr L . Where the heart is: Meeting the psychosocial needs of young children in the context of HIV/AIDS. The Hague, The Netherlands: Bernard van Leer Foundation.

[58] England MJ , Butler AS , Gonzalez ML . Psychosocial interventions for mental and substance use disorders: A framework for establishing evidence‐based standards. 2015. 26203478

[59] Veritas Health Innovation . Covidence systematic review software. Melbourne, Australia: Veritas; 2020.

[60] Higgins JPT , Altman DG , Gotzsche PC , Juni P , Moher D , Oxman AD , et al. The Cochrane Collaboration’s tool for assessing risk of bias in randomised trials. BMJ. 2011;343:d5928. 22008217 10.1136/bmj.d5928 PMC3196245

[61] Gradepro GDT . GRADEpro guideline development tool [software]. Hamilton: McMaster University; 2015.

[62] World Health Organization . mhGAP intervention guide for mental, neurological and substance use disorders in non‐specialized health settings: mental health Gap Action Programme (mhGAP). Geneva: World Health Organization; 2016. 27786430

[63] Bermudez LG , Ssewamala FM , Neilands TB , Lu L , Jennings L , Nakigozi G , et al. Does economic strengthening improve viral suppression among adolescents living with HIV? Results from a cluster randomized trial in Uganda. AIDS and Behavior. 2018;22(11):3763–72. 29846836 10.1007/s10461-018-2173-7 PMC6204092

[64] Lightfoot MA , Kasirye R , Comulada WS , Rotheram‐Borus MJ . Efficacy of a culturally adapted intervention for youth living with HIV in Uganda. Prevent Sci. 2007;8(4):271–3. 10.1007/s11121-007-0074-5 PMC2819813 17846891

[65] Linnemayr S , Huang H , Luoto J , Kambugu A , Thirumurthy H , Haberer JE , et al. Text messaging for improving antiretroviral therapy adherence: no effects after 1 year in a randomized controlled trial among adolescents and young. Adults. 2017;107(12):1944–50. 10.2105/AJPH.2017.304089 PMC5678388 29048966

[66] MacCarthy S , Wagner Z , Mendoza‐Graf A , Gutierrez CI , Samba C , Birungi J , et al. A randomized controlled trial study of the acceptability, feasibility, and preliminary impact of SITA (SMS as an Incentive To Adhere): a mobile technology‐based intervention informed by behavioral economics to improve ART adherence among youth in Uganda. BMC Infect Dis. 2020;20(1):173. 32093630 10.1186/s12879-020-4896-0 PMC7041095

[67] Ssewamala FM , Dvalishvili D , Mellins CA , Geng EH , Makumbi F , Neilands TB , et al. The long‐term effects of a family based economic empowerment intervention (Suubi+Adherence) on suppression of HIV viral loads among adolescents living with HIV in southern Uganda: Findings from 5‐year cluster randomized trial. PLOS ONE. 2020;15:e0228370. 32040523 10.1371/journal.pone.0228370 PMC7010288

[68] Nestadt DF , Saisaengjan C , McKay MM , Bunupuradah T , Pardo G , Lakhonpon S , et al. CHAMP+ Thailand: pilot randomized control trial of a family‐based psychosocial intervention for perinatally HIV‐infected early. Adolescents. 2019;33(5):227–36. 10.1089/apc.2019.0021 PMC6531900 31067121

[69] Rongkavilit C , Naar‐King S , Wang B , Panthong A , Bunupuradah T , Parsons JT , et al. Motivational interviewing targeting risk behaviors for youth living with HIV in Thailand. AIDS Behav. 2013;17(6):2063–74. 23325376 10.1007/s10461-013-0407-2 PMC3669670

[70] Rongkavilit C , Wang B , Naar‐King S , Bunupuradah T , Parsons JT , Panthong A , et al. Motivational interviewing targeting risky sex in HIV‐positive young thai men who have sex with men. Arch Sex Behav. 2015;44(2):329–40. 24668304 10.1007/s10508-014-0274-6 PMC4177013

[71] Kaihin R , Kasatpibal N , Chitreechuer J , Grimes RM . Effect of an empowerment intervention on antiretroviral drug adherence in Thai youth. Behav Med. 2015;41(4):186–94. 24758271 10.1080/08964289.2014.911717 PMC4375063

[72] Mavhu W , Willis N , Mufuka J , Bernays S , Tshuma M , Mangenah C , et al. Effect of a differentiated service delivery model on virological failure in adolescents with HIV in Zimbabwe (Zvandiri): a cluster‐randomised controlled trial. The Lancet Global Health. 2020;8(2):e264–75. 31924539 10.1016/S2214-109X(19)30526-1

[73] Willis N , Milanzi A , Mawodzeke M , Dziwa C , Armstrong A , Yekeye I , et al. Effectiveness of community adolescent treatment supporters (CATS) interventions in improving linkage and retention in care, adherence to ART and psychosocial well‐being: a randomised trial among adolescents living with HIV in rural Zimbabwe. BMC Public Health. 2019;19(1):117. 30691425 10.1186/s12889-019-6447-4 PMC6348677

[74] Dulli L , Ridgeway K , Packer C , Murray KR , Mumuni T , Plourde KF , et al. A social media‐based support group for youth living with HIV in Nigeria (SMART Connections): randomized controlled trial. J Med Internet Res. 2020;22:e18343. 32484444 10.2196/18343 PMC7298637

[75] Bhana A , Mellins CA , Petersen I , Alicea S , Myeza N , Holst H , et al. The VUKA family program: piloting a family‐based psychosocial intervention to promote health and mental health among HIV infected early adolescents in South Africa. AIDS Care. 2014;26(1):1–11. 23767772 10.1080/09540121.2013.806770 PMC3838445

[76] Denison JA , Burke VM , Miti S , Nonyane BA , Frimpong C , Merrill KG , et al., Project YES! Youth Engaging for Success: a randomized controlled trial assessing the impact of a clinic‐based peer mentoring program on viral suppression, adherence and internalized stigma among HIV‐positive youth (15‐24 years) in Ndola, Zambia. PLoS One. 2020. 15:e0230703. 32240186 10.1371/journal.pone.0230703 PMC7117673

[77] Brown LK , Kennard BD , Emslie GJ , Mayes TL , Whiteley LB , Bethel J , et al. Effective treatment of depressive disorders in medical clinics for adolescents and young adults living with HIV. JAIDS J Acquir Immune Defic Syndr. 2016;71(1):38–46. 26761270 10.1097/QAI.0000000000000803 PMC4712723

[78] Bouris A , Jaffe K , Eavou R , Liao C , Kuhns L , Voisin D , et al. Project nGage: results of a randomized controlled trial of a dyadic network support intervention to retain young black men who have sex with men in HIV care. AIDS Behav. 2017;21(12):3618–29. 29079949 10.1007/s10461-017-1954-8 PMC5705428

[79] Brothers J , Hotton AL , Hosek SG , Harper GW , Fernandez MI . Young women living with HIV: outcomes from a targeted secondary prevention empowerment pilot trial. AIDS Patient Care STDs. 2016;30(5):229–35. 27158851 10.1089/apc.2015.0294 PMC4870604

[80] Jones DL , Echenique M , Potter J , Rodriguez VJ , Weiss SM , Fischl MA Adolescent girls and young women living with HIV: preconception counseling strategies. Int J Womens Health. 2017;9:657–63. 29066934 10.2147/IJWH.S136668 PMC5605185

[81] Spratt ES , Papa CE , Mueller M , Patel S , Killeen T , Maher E , et al. Using technology to improve adherence to HIV medications in transitional age youth: research reviewed, methods tried, lessons learned. J Gen Med (Dover). 2017;1(1):1002. 30345429 PMC6195351

[82] Naar‐King S , Outlaw AY , Sarr M , Parsons JT , Belzer M , MacDonell K , et al. Motivational enhancement system for adherence (MESA): pilot randomized trial of a brief computer‐delivered prevention intervention for youth initiating antiretroviral treatment. J Pediatric Psychol. 2013;38(6):638–48. 10.1093/jpepsy/jss132 PMC3701125 23359664

[83] Berrien VM , Salazar JC , Reynolds E , Mckay K . Adherence to antiretroviral therapy in HIV‐infected pediatric patients improves with home‐based intensive nursing intervention. AIDS Patient Care STDs. 2004;18(6):355–63. 15294086 10.1089/1087291041444078

[84] Garofalo R , Kuhns LM , Hotton A , Johnson A , Muldoon A , Rice D . A randomized controlled trial of personalized text message reminders to promote medication adherence among HIV‐positive adolescents and young adults. AIDS Behav. 2016;20(5):1049–59. 26362167 10.1007/s10461-015-1192-x PMC4788595

[85] Whiteley L , Brown LK , Mena L , Craker L , Arnold T . Enhancing health among youth living with HIV using an iPhone game. AIDS Care. 2018;30 Sup4:21–33. 30626196 10.1080/09540121.2018.1503224 PMC6422754

[86] Belzer ME , Naar‐King S , Olson J , Sarr M , Thornton S , Kahana SY , et al. The use of cell phone support for non‐adherent HIV‐infected youth and young adults: an initial randomized and controlled intervention trial. AIDS Behav. 2014;18(4):686–96. 24271347 10.1007/s10461-013-0661-3 PMC3962719

[87] Sayegh CS , MacDonell KK , Clark LF , Dowshen NL , Naar S , Olson‐Kennedy J , et al. The impact of cell phone support on psychosocial outcomes for youth living with HIV nonadherent to antiretroviral therapy. AIDS Behav. 2018;22(10):3357–62. 29948339 10.1007/s10461-018-2192-4 PMC6530981

[88] Chen X , Murphy DA , Naar‐King S , Parsons JT . A clinic‐based motivational intervention improves condom use among subgroups of youth living with HIV. J Adolesc Health. 2011;49(2):193–8. 21783053 10.1016/j.jadohealth.2010.11.252 PMC3282587

[89] Naar‐King S , Parsons JT , Murphy DA , Chen X , Harris DR , Belzer ME . Improving health outcomes for youth living with the human immunodeficiency virus. Arch Pediatr Adolesc Med. 2009;163(12):1092–8. 19996045 10.1001/archpediatrics.2009.212 PMC2843389

[90] Mimiaga MJ , Bogart LM , Thurston IB , Santostefano CM , Closson EF , Skeer MR , et al. Positive strategies to enhance problem‐solving skills (STEPS): a pilot randomized, controlled trial of a multicomponent, technology‐enhanced, customizable antiretroviral adherence intervention for HIV‐infected adolescents and young adults. AIDS Patient Care STDS. 2019;33(1):21–4. 30601059 10.1089/apc.2018.0138 PMC6338456

[91] Naar‐King S , Wright K , Parsons JT , Frey M , Templin T , Lam P , et al. Healthy choices: motivational enhancement therapy for health risk behaviors in HIV‐positive youth. AIDS Educ Prevent. 2006;18(1):1–11. 10.1521/aeap.2006.18.1.1 16539572

[92] Naar‐King S , Lam P , Wang B , Wright K , Parsons JT , Frey MA . Brief report: maintenance of effects of motivational enhancement therapy to improve risk behaviors and HIV‐related health in a randomized controlled trial of youth living with HIV. J Pediatr Psychol. 2007;33(4):441–5. 17905800 10.1093/jpepsy/jsm087

[93] Hosek SG , Harper GW , Lemos D , Burke‐Miller J , Lee S , Friedman L , et al. Project ACCEPT: evaluation of a group‐based intervention to improve engagement in care for youth newly diagnosed with HIV. AIDS and Behav. 2018;22(8):2650–61. 10.1007/s10461-018-2034-4 29396633

[94] Webb L , Perry‐Parrish C , Ellen J , Sibinga E . Mindfulness instruction for HIV‐infected youth: a randomized controlled trial. AIDS Care. 2018;30(6):688–95. 29067834 10.1080/09540121.2017.1394434 PMC5987527

[95] Christodoulou J , Abdalian SE , Jones ASK , Christodoulou G , Pentoney SL , Rotheram‐Borus MJ . Crystal clear with active visualization: understanding medication adherence among youth living with HIV. AIDS Behav. 2020;24(4):1207–11. 31696369 10.1007/s10461-019-02721-3 PMC7085439

[96] Rotheram‐Borus MJ , Swendeman D , Comulada WS , Weiss RE , Lee M , Lightfoot M . Prevention for substance‐using HIV‐positive young people. JAIDS J Acquir Immune Defic Syndr. 2004;37 Supplement 2:S68–77. 15385902 10.1097/01.qai.0000140604.57478.67 PMC2843590

[97] Murray E , Burns J , See Tai S , Lai R , Nazareth I . Interactive health communication applications for people with chronic disease. Cochrane Database Syst Rev. 2005;4:CD004274. 10.1002/14651858.CD004274.pub4 PMC13184810 16235356

[98] Naar‐King S , Parsons JT , Murphy D , Kolmodin K , Harris DR . A Multisite randomized trial of a motivational intervention targeting multiple risks in youth living with HIV: initial effects on motivation, self‐efficacy, and depression. J Adolesc Health. 2010;46(5):422–8. 20413077 10.1016/j.jadohealth.2009.11.198 PMC2859210

[99] Archary M , Pettifor AE , Toska E . Adolescents and young people at the centre: global perspectives and approaches to transform HIV testing, treatment and care. J Int AIDS Soc. 2020;23:e25581. 32869490 10.1002/jia2.25581 PMC7459165

[100] Murray KR , Dulli LS , Ridgeway K , Dal Santo L , Darrow de Mora D , Olsen P , et al. Improving retention in HIV care among adolescents and adults in low‐ and middle‐income countries: a systematic review of the literature. PLoS One. 2017;12:e0184879. 28961253 10.1371/journal.pone.0184879 PMC5621671

[101] Casale M , Carlqvist A , Cluver L . Recent interventions to improve retention in HIV care and adherence to antiretroviral treatment among adolescents and youth: a systematic review. AIDS Patient Care STDS. 2019;33(6):237–52. 31166783 10.1089/apc.2018.0320 PMC6588099

[102] Mwale M , Muula AS . Systematic review: a review of adolescent behavior change interventions [BCI] and their effectiveness in HIV and AIDS prevention in sub‐Saharan Africa. BMC Public Health. 2017;17(1):718. 28923040 10.1186/s12889-017-4729-2 PMC5604191

[103] Muthoni CN , Kneipp SM , Gichane MW , Caiola CE , Pettifor AE , Williams JR . A Systematic review of hiv interventions for young women in Sub‐Saharan Africa. AIDS Behav. 2020;24(12):3395–413. 32410052 10.1007/s10461-020-02914-1

[104] Ridgeway K , Dulli LS , Murray KR , Silverstein H , Dal Santo L , Olsen P , Darrow de Mora D , et al. Interventions to improve antiretroviral therapy adherence among adolescents in low‐ and middle‐income countries: a systematic review of the literature. PLoS One. 2018;13:e0189770. 29293523 10.1371/journal.pone.0189770 PMC5749726

[105] Delgado‐Rodriguez M , Llorca J . Bias. J Epidemiol Commun Health. 2004;58(8):635–41. 10.1136/jech.2003.008466 PMC1732856 15252064

[106] Bouris A , Jaffe K , Eavou R , Liao C , Kuhns L , Voisin D , et al. Project nGage: results of a randomized controlled trial of a dyadic network support intervention to retain young black men who have sex with men in HIV care. AIDS Behav. 2017;21(12):3618–29. 29079949 10.1007/s10461-017-1954-8 PMC5705428

[107] Nestadt DF , Saisaengjan C , McKay MM , Bunupuradah T , Pardo G , Lakhonpon S , et al. CHAMP+ Thailand: pilot randomized control trial of a family‐based psychosocial intervention for perinatally HIV‐infected early adolescents. AIDS Patient Care STDS. 2019;33(5):227–36. 31067121 10.1089/apc.2019.0021 PMC6531900

[108] Horwood C , Butler LM , Haskins L , Phakathi S , Rollins N . HIV‐infected adolescent mothers and their infants: low coverage of HIV services and high risk of HIV transmission in KwaZulu‐Natal, South Africa. PLoS ONE. 2013;8:e74568. 24073215 10.1371/journal.pone.0074568 PMC3779214

[109] Toska E , Cluver L , Laurenzi CA , Wittesaele C , Sherr L , Zhou S , et al. Reproductive aspirations, contraception use and dual protection among adolescent girls and young women: the effect of motherhood and HIV status. J Int AIDS Soc. 2020;23:e25558. 32869543 10.1002/jia2.25558 PMC7459160

[110] Belle JA , Gamedze NN . Behavioral factors contributing to the transmission of HIV and AIDS amongst young women of Mbabane in Swaziland. Afr Health Sci. 2019;19(3):2302–11. 32127799 10.4314/ahs.v19i3.2 PMC7040258

[111] Toska E , Pantelic M , Meinck F , Keck K , Haghighat R , Cluver L . Sex in the shadow of HIV: a systematic review of prevalence, risk factors, and interventions to reduce sexual risk‐taking among HIV‐positive adolescents and youth in sub‐Saharan Africa. PLoS One. 2017;12:e0178106. 28582428 10.1371/journal.pone.0178106 PMC5459342

[112] Desrosiers A , Betancourt T , Kergoat Y , Servilli C , Say L , Kobeissi L , et al. A systematic review of sexual and reproductive health interventions for young people in humanitarian and lower‐and‐middle‐income country settings. BMC Public Health. 2020;20(1):666. 32398129 10.1186/s12889-020-08818-y PMC7216726

[113] Mavegam BO , Pharr JR , Cruz P , Ezeanolue EE . Effective interventions to improve young adults' linkage to HIV care in Sub‐Saharan Africa: a systematic review. AIDS Care. 2017;29(10):1198–204. 28325077 10.1080/09540121.2017.1306637

[114] Ryscavage P , Macharia T , Patel D , Palmeiro R , Tepper V . Linkage to and retention in care following healthcare transition from pediatric to adult HIV care. AIDS Care. 2016;28(5):561–5. 26766017 10.1080/09540121.2015.1131967

[115] Sohn AH , Vreeman RC , Judd A . Tracking the transition of adolescents into adult HIV care: a global assessment. J Int AIDS Soc. 2017;20:21878. 28530035 10.7448/IAS.20.4.21878 PMC5577733

[116] Wong VJ , Murray KR , Phelps BR , Vermund SH , McCarraher DR . Adolescents, young people, and the 90–90‐90 goals: a call to improve HIV testing and linkage to treatment. AIDS. 2017;31 Suppl 3:S191–4. 28665876 10.1097/QAD.0000000000001539 PMC5497776

[117] Mimiaga MJ , Bogart LM , Thurston IB , Santostefano CM , Closson EF , Skeer MR , et al. Positive strategies to enhance problem‐solving skills (STEPS): a pilot randomized, controlled trial of a multicomponent, technology‐enhanced, customizable antiretroviral adherence intervention for hiv‐infected adolescents and young adults. AIDS Patient Care STDS. 2019;33(1):21–4. 30601059 10.1089/apc.2018.0138 PMC6338456

[118] Whiteley L , Brown LK , Mena L , Craker L , Arnold T . Enhancing health among youth living with HIV using an iPhone game. AIDS Care. 2018;30 Sup4:21–33. 30626196 10.1080/09540121.2018.1503224 PMC6422754

[119] Brothers J , Hotton AL , Hosek SG , Harper GW , Fernandez MI . Young women living with HIV: outcomes from a targeted secondary prevention empowerment pilot trial. AIDS Patient Care STDS. 2016;30(5):229–35. 27158851 10.1089/apc.2015.0294 PMC4870604

[120] Lightfoot MA , Kasirye R , Comulada WS , Rotheram‐Borus MJ . Efficacy of a culturally adapted intervention for youth living with HIV in Uganda. Prev Sci. 2007;8(4):271–3. 17846891 10.1007/s11121-007-0074-5 PMC2819813

[121] Linnemayr S , Huang H , Luoto J , Kambugu A , Thirumurthy H , Haberer JE , et al. Text messaging for improving antiretroviral therapy adherence: no effects after 1 year in a randomized controlled trial among adolescents and young adults. Am J Public Health. 2017;107(12):1944–50. 29048966 10.2105/AJPH.2017.304089 PMC5678388

[122] Garofalo R , Kuhns LM , Hotton A , Johnson A , Muldoon A , Rice D . A randomized controlled trial of personalized text message reminders to promote medication adherence among HIV‐positive adolescents and young adults. AIDS Behav. 2016;20(5):1049–59. 26362167 10.1007/s10461-015-1192-x PMC4788595

[123] Denison JA , Burke VM , Miti S , Nonyane BAS , Frimpong C , Merrill KG , et al. Project YES! youth engaging for success: a randomized controlled trial assessing the impact of a clinic‐based peer mentoring program on viral suppression, adherence and internalized stigma among HIV‐positive youth (15–24 years) in Ndola, Zambia. PLoS One. 2020;15:e0230703. 32240186 10.1371/journal.pone.0230703 PMC7117673

[124] Laurenzi CA , Skeen S , Gordon S , Akin‐Olugbade O , Abrahams N , Bradshaw M , et al. Preventing mental health conditions in adolescents living with HIV: an urgent need for evidence. J Int AIDS Soc. 2020;23:e25556. 32869530 10.1002/jia2.25556 PMC7459172

[125] Wingood GM , DiClemente RJ . The ADAPT‐ITT model: a novel method of adapting evidence‐based HIV Interventions. J Acquir Immune Defic Syndr. 2008;47 Suppl 1:S40–6. 18301133 10.1097/QAI.0b013e3181605df1

[126] McKleroy VS , Galbraith JS , Cummings B , Jones P , Harshbarger C , Collins C , et al. Adapting evidence–based behavioral interventions for new settings and target populations. AIDS Educ Prevent. 2006;18 Supp:59–73. 10.1521/aeap.2006.18.supp.59 16987089

[127] Movsisyan A , Arnold L , Evans R , Hallingberg B , Moore G , O’Cathain A , et al. Adapting evidence‐informed complex population health interventions for new contexts: a systematic review of guidance. Implement Sci. 2019;14(1):105. 31847920 10.1186/s13012-019-0956-5 PMC6918624

[128] Canning D . The economics of HIV/AIDS in low‐income countries: the case for prevention. J Econom Perspect. 2006;20(3):121–42. 10.1257/jep.20.3.121 17176527

[129] Hudelson C , Cluver L . Factors associated with adherence to antiretroviral therapy among adolescents living with HIV/AIDS in low‐and middle‐income countries: a systematic review. AIDS Care. 2015;27(7):805–16. 25702789 10.1080/09540121.2015.1011073

[130] Busza J , Dauya E , Bandason T , Simms V , Chikwari CD , Makamba M , et al. The role of community health workers in improving HIV treatment outcomes in children: lessons learned from the ZENITH trial in Zimbabwe. Health Policy Plan. 2018;33(3):328–34. 29309578 10.1093/heapol/czx187 PMC5886269

[131] Oliveras C , Cluver L , Bernays S , Armstrong A . Nothing about us without RIGHTS—meaningful engagement of children and youth: from research prioritization to clinical trials, implementation science, and policy. J Acquir Immune Defic Syndr. 2018;78(1):S27. 29994917 10.1097/QAI.0000000000001746 PMC6075896

[132] UNICEF . Prioritizing the Continuity of Services for Adolescents Living with HIV During the COVID‐19 Pandemic, UNICEF and WHO Adolescent Service Delivery Working Group, Editors. 5 June 2020.

[133] Vrazo AC , Golin R , Fernando NB , Killam WP , Sharifi S , Phelps B , et al. Adapting HIV services for pregnant and breastfeeding women, infants, children, adolescents and families in resource‐constrained settings during the COVID‐19 pandemic. J Int AIDS Soc. 2020;23:e25622. 32996705 10.1002/jia2.25622 PMC7525801

[134] Paediatric‐Adolescent Treatment Africa (PATA) . Evidence Brief: Caring during COVID‐19: supporting mental health among vulnerable adolescents and young people. Cape Town; 2020.

[135] Hightow‐Weidman L , Muessig K , Claude K , Roberts J , Zlotorzynska M , Sanchez T , et al. Maximizing digital interventions for youth in the midst of COVID‐19: lessons from the adolescent trials network for HIV interventions. AIDS Behav. 2020;24(8):2239–43. 32306214 10.1007/s10461-020-02870-w PMC7166094

[136] Celik R , Toruner EK . The effect of technology‐based programmes on changing health behaviours of adolescents: systematic review. Compr Child Adolesc Nurs. 2020;43(2):92–110. 31157992 10.1080/24694193.2019.1599083

[137] Palmer MJ , Henschke N , Villanueva G , Maayan N , Bergman H , Glenton C , et al. Targeted client communication via mobile devices for improving sexual and reproductive health. Cochrane Database Syst Rev. 2020;8:CD013680. 32779730 10.1002/14651858.CD013680 PMC8409381

[138] Ames HM , Glenton C , Lewin S , Tamrat T , Akama E , Leon N . Clients' perceptions and experiences of targeted digital communication accessible via mobile devices for reproductive, maternal, newborn, child, and adolescent health: a qualitative evidence synthesis. Cochrane Database Syst Rev. 2019;10:CD013447. 31608981 10.1002/14651858.CD013447 PMC6791116

