#  

**DOI:** 10.1002/jia2.26014

**Published:** 2022-09-07

In the article [1], there was an error made in the Table 1. On page 6, Mimiaga et al. (2019) have the incorrect *n* value.

The sentence reads: “Young people living with HIV (*n* = 60)”

The sentence should have read: “Young people living with HIV (*n* = 14)”
