# Supplementary material for: Psychosocial interventions for improving engagement in care and health and behavioural outcomes for adolescents and young people living with HIV: a systematic review and meta‐analysis
Source: J Int AIDS Soc. 2021 Aug 2;24(8):e25741. doi: 10.1002/jia2.25741 (PMC8327356; doi:10.1002/jia2.25741)
Supplement: Supplementary file 1 — Table S1. Search strategy [file JIA2-24-e25741-s003.docx]

**Additional File 1: Search strategy**

**Title: For adolescents and young people living with HIV, should psychosocial interventions be considered to improve engagement in care and other health outcomes**

**Database: Scopus**

**Date: 1 July 2020**

|  | **Query** | **Results** |
| --- | --- | --- |
| 13 | ((TITLE-ABS-KEY("human immunodeficiency virus infection" OR "human immunodeficiency virus" OR "human immuno deficiency virus" OR "human immunedeficiency virus" OR "human immune deficiency virus" OR hiv OR "hiv-1" OR "hiv-2" OR "acquired immunodeficiency syndrome" OR "acquired immuno deficiency syndrome" OR "acquired immunedeficiency syndrome" OR "acquired immune deficiency syndrome" )) AND (TITLE-ABS-KEY(adolescen* OR teen OR teens OR teenage OR teenager* OR youth* OR "young adult*" OR "young person*" OR "young people" OR "young women" OR "young females" OR "young men" OR "young males" OR youngster* OR juvenil* OR student* OR pupils OR "young adulthood" )) AND ((TITLE-ABS-KEY("psychosocial support" OR "social support" OR "self help" OR psychotherapy OR counselling OR counseling OR "cognitive behavioral therapy" OR "adaptive behavior" OR "health education" OR "behavior therapy" OR mindfulness OR "psychological aspect" )) OR ((TITLE-ABS-KEY(psychology OR psychological OR psychosocial OR socio* OR social* OR educat* OR behavio* OR cope OR coping OR cognitive OR cognition OR psychoeducat* OR interpersonal OR "problem-solv*" OR network* OR family OR families OR community OR communities OR group OR groups OR peer OR "self-help" OR selfhelp )) AND (TITLE-ABS-KEY(intervention* OR therap* OR program* OR support OR strateg* OR skill* OR counsel* OR technique* ))))) AND ((INDEXTERMS ("randomized controlled trial" OR "crossover procedure" OR "double-blind procedure" OR "single-blind procedure")) OR (TITLE-ABS("randomized controlled trial" OR random* OR trial OR allocat* OR factorial* OR placebo* OR assign* OR volunteer* OR "crossover procedure" OR "double-blind procedure" OR "single-blind procedure" OR (doubl* W/ 3 blind*) OR (singl* AND blind*) OR crossover* OR "cross+over*" OR (cross W/ 1 over*) ))) AND PUBYEAR > 1999 | [5,947 document results](https://www.scopus.com/search/history/results.uri?origin=searchhistory&shid=13) |
| 12 | ( ( TITLE-ABS-KEY ( "human immunodeficiency virus infection" OR "human immunodeficiency virus" OR "human immuno deficiency virus" OR "human immunedeficiency virus" OR "human immune deficiency virus" OR hiv OR "hiv-1" OR "hiv-2" OR "acquired immunodeficiency syndrome" OR "acquired immuno deficiency syndrome" OR "acquired immunedeficiency syndrome" OR "acquired immune deficiency syndrome" ) ) AND ( TITLE-ABS-KEY ( adolescen* OR teen OR teens OR teenage OR teenager* OR youth* OR "young adult*" OR "young person*" OR "young people" OR "young women" OR "young females" OR "young men" OR "young males" OR youngster* OR juvenil* OR student* OR pupils OR "young adulthood" ) ) AND ( ( TITLE-ABS-KEY ( "psychosocial support" OR "social support" OR "self help" OR psychotherapy OR counselling OR counseling OR "cognitive behavioral therapy" OR "adaptive behavior" OR "health education" OR "behavior therapy" OR mindfulness OR "psychological aspect" ) ) OR ( ( TITLE-ABS-KEY ( psychology OR psychological OR psychosocial OR socio* OR social* OR educat* OR behavio* OR cope OR coping OR cognitive OR cognition OR psychoeducat* OR interpersonal OR "problem-solv*" OR network* OR family OR families OR community OR communities OR group OR groups OR peer OR "self-help" OR selfhelp ) ) AND ( TITLE-ABS-KEY ( intervention* OR therap* OR program* OR support OR strateg* OR skill* OR counsel* OR technique* ) ) ) ) ) AND ( ( INDEXTERMS ( "randomized controlled trial" OR "crossover procedure" OR "double-blind procedure" OR "single-blind procedure" ) ) OR ( TITLE-ABS ( "randomized controlled trial" OR random* OR trial OR allocat* OR factorial* OR placebo* OR assign* OR volunteer* OR "crossover procedure" OR "double-blind procedure" OR "single-blind procedure" OR ( doubl* W/ 3 blind* ) OR ( singl* AND blind* ) OR crossover* OR "cross+over*" OR ( cross W/ 1 over* ) ) ) ) | [6,679 document results](https://www.scopus.com/search/history/results.uri?origin=searchhistory&shid=12) |
| 11 | ( INDEXTERMS ( "randomized controlled trial" OR "crossover procedure" OR "double-blind procedure" OR "single-blind procedure" ) ) OR ( TITLE-ABS ( "randomized controlled trial" OR random* OR trial OR allocat* OR factorial* OR placebo* OR assign* OR volunteer* OR "crossover procedure" OR "double-blind procedure" OR "single-blind procedure" OR ( doubl* W/ 3 blind* ) OR ( singl* AND blind* ) OR crossover* OR "cross+over*" OR ( cross W/ 1 over* ) ) ) | [4,485,918 document results](https://www.scopus.com/search/history/results.uri?origin=searchhistory&shid=11) |
| 10 | TITLE-ABS ( "randomized controlled trial" OR random* OR trial OR allocat* OR factorial* OR placebo* OR assign* OR volunteer* OR "crossover procedure" OR "double-blind procedure" OR "single-blind procedure" OR ( doubl* W/ 3 blind* ) OR ( singl* AND blind* ) OR crossover* OR "cross+over*" OR ( cross W/ 1 over* ) ) | [4,307,013 document results](https://www.scopus.com/search/history/results.uri?origin=searchhistory&shid=10) |
| 9 | INDEXTERMS ( "randomized controlled trial" OR "crossover procedure" OR "double-blind procedure" OR "single-blind procedure" ) | [764,820 document results](https://www.scopus.com/search/history/results.uri?origin=searchhistory&shid=9) |
| 8 | ( TITLE-ABS-KEY ( "human immunodeficiency virus infection" OR "human immunodeficiency virus" OR "human immuno deficiency virus" OR "human immunedeficiency virus" OR "human immune deficiency virus" OR hiv OR "hiv-1" OR "hiv-2" OR "acquired immunodeficiency syndrome" OR "acquired immuno deficiency syndrome" OR "acquired immunedeficiency syndrome" OR "acquired immune deficiency syndrome" ) ) AND ( TITLE-ABS-KEY ( adolescen* OR teen OR teens OR teenage OR teenager* OR youth* OR "young adult*" OR "young person*" OR "young people" OR "young women" OR "young females" OR "young men" OR "young males" OR youngster* OR juvenil* OR student* OR pupils OR "young adulthood" ) ) AND ( ( TITLE-ABS-KEY ( "psychosocial support" OR "social support" OR "self help" OR psychotherapy OR counselling OR counseling OR "cognitive behavioral therapy" OR "adaptive behavior" OR "health education" OR "behavior therapy" OR mindfulness OR "psychological aspect" ) ) OR ( ( TITLE-ABS-KEY ( psychology OR psychological OR psychosocial OR socio* OR social* OR educat* OR behavio* OR cope OR coping OR cognitive OR cognition OR psychoeducat* OR interpersonal OR "problem-solv*" OR network* OR family OR families OR community OR communities OR group OR groups OR peer OR "self-help" OR selfhelp ) ) AND ( TITLE-ABS-KEY ( intervention* OR therap* OR program* OR support OR strateg* OR skill* OR counsel* OR technique* ) ) ) ) | [37,642 document results](https://www.scopus.com/search/history/results.uri?origin=searchhistory&shid=8) |
| 7 | ( TITLE-ABS-KEY ( "psychosocial support" OR "social support" OR "self help" OR psychotherapy OR counselling OR counseling OR "cognitive behavioral therapy" OR "adaptive behavior" OR "health education" OR "behavior therapy" OR mindfulness OR "psychological aspect" ) ) OR ( ( TITLE-ABS-KEY ( psychology OR psychological OR psychosocial OR socio* OR social* OR educat* OR behavio* OR cope OR coping OR cognitive OR cognition OR psychoeducat* OR interpersonal OR "problem-solv*" OR network* OR family OR families OR community OR communities OR group OR groups OR peer OR "self-help" OR selfhelp ) ) AND ( TITLE-ABS-KEY ( intervention* OR therap* OR program* OR support OR strateg* OR skill* OR counsel* OR technique* ) ) ) | [7,897,962 document results](https://www.scopus.com/search/history/results.uri?origin=searchhistory&shid=7) |
| 6 | ( TITLE-ABS-KEY ( psychology OR psychological OR psychosocial OR socio* OR social* OR educat* OR behavio* OR cope OR coping OR cognitive OR cognition OR psychoeducat* OR interpersonal OR "problem-solv*" OR network* OR family OR families OR community OR communities OR group OR groups OR peer OR "self-help" OR selfhelp ) ) AND ( TITLE-ABS-KEY ( intervention* OR therap* OR program* OR support OR strateg* OR skill* OR counsel* OR technique* ) ) | [7,427,142 document results](https://www.scopus.com/search/history/results.uri?origin=searchhistory&shid=6) |
| 5 | TITLE-ABS-KEY ( intervention* OR therap* OR program* OR support OR strateg* OR skill* OR counsel* OR technique* ) | [20,849,276 document results](https://www.scopus.com/search/history/results.uri?origin=searchhistory&shid=5) |
| 4 | TITLE-ABS-KEY ( psychology OR psychological OR psychosocial OR socio* OR social* OR educat* OR behavio* OR cope OR coping OR cognitive OR cognition OR psychoeducat* OR interpersonal OR "problem-solv*" OR network* OR family OR families OR community OR communities OR group OR groups OR peer OR "self-help" OR selfhelp ) | [21,300,394 document results](https://www.scopus.com/search/history/results.uri?origin=searchhistory&shid=4) |
| 3 | TITLE-ABS-KEY ( "psychosocial support" OR "social support" OR "self help" OR psychotherapy OR counselling OR counseling OR "cognitive behavioral therapy" OR "adaptive behavior" OR "health education" OR "behavior therapy" OR mindfulness OR "psychological aspect" ) | [1,162,971 document results](https://www.scopus.com/search/history/results.uri?origin=searchhistory&shid=3) |
| 2 | TITLE-ABS-KEY ( adolescen* OR teen OR teens OR teenage OR teenager* OR youth* OR "young adult*" OR "young person*" OR "young people" OR "young women" OR "young females" OR "young men" OR "young males" OR youngster* OR juvenil* OR student* OR pupils OR "young adulthood" ) | [4,230,474 document results](https://www.scopus.com/search/history/results.uri?origin=searchhistory&shid=2) |
| 1 | TITLE-ABS-KEY ( "human immunodeficiency virus infection" OR "human immunodeficiency virus" OR "human immuno deficiency virus" OR "human immunedeficiency virus" OR "human immune deficiency virus" OR hiv OR "hiv-1" OR "hiv-2" OR "acquired immunodeficiency syndrome" OR "acquired immuno deficiency syndrome" OR "acquired immunedeficiency syndrome" OR "acquired immune deficiency syndrome" ) | [516,619 document results](https://www.scopus.com/search/history/results.uri?origin=searchhistory&shid=1) |

**Database: PubMed**

**Date: 30 June 2020**

| **Search** | **Query** | **Results** |
| --- | --- | --- |
| #11 | Search: **(#8 AND #9) AND (("2000/01/01"[Date - Publication] : "2020/06/30"[Date - Publication]))** | [9,673](https://pubmed.ncbi.nlm.nih.gov/?term=%28%238+AND+%239%29+AND+%28%28%222000%2F01%2F01%22%5BDate+-+Publication%5D+%3A+%222020%2F06%2F30%22%5BDate+-+Publication%5D%29%29&sort=relevance&size=200&ac=no) |
| #10 | Search: **#8 AND #9** | [11,045](https://pubmed.ncbi.nlm.nih.gov/?term=%238+AND+%239&sort=relevance&size=200&ac=no) |
| #9 | Search: **(randomized controlled trial [pt] OR controlled clinical trial [pt] OR randomized [tiab] OR placebo [tiab] OR drug therapy [sh] OR randomly [tiab] OR trial [tiab] OR groups [tiab]) NOT (animals [mh] NOT humans [mh])** | [4,164,967](https://pubmed.ncbi.nlm.nih.gov/?term=%28randomized+controlled+trial+%5Bpt%5D+OR+controlled+clinical+trial+%5Bpt%5D+OR+randomized+%5Btiab%5D+OR+placebo+%5Btiab%5D+OR+drug+therapy+%5Bsh%5D+OR+randomly+%5Btiab%5D+OR+trial+%5Btiab%5D+OR+groups+%5Btiab%5D%29+NOT+%28animals+%5Bmh%5D+NOT+humans+%5Bmh%5D%29&sort=relevance&size=200&ac=no) |
| #8 | Search: **#1 AND #2 AND #7** | [25,505](https://pubmed.ncbi.nlm.nih.gov/?term=%231+AND+%232+AND+%237&sort=relevance&size=200&ac=no) |
| #7 | Search: **#3 OR #6** | [2,670,912](https://pubmed.ncbi.nlm.nih.gov/?term=%233+OR+%236&sort=relevance&size=200&ac=no) |
| #6 | Search: **#4 AND #5** | [2,422,890](https://pubmed.ncbi.nlm.nih.gov/?term=%234+AND+%235&sort=relevance&size=200&ac=no) |
| #5 | Search: **psychology[tiab] OR psychological[tiab] OR psychosocial[tiab] OR psychotherapy[tiab] OR socio*[tiab] OR social*[tiab] OR educat*[tiab] OR behavio*[tiab] OR cope[tiab] OR coping[tiab] OR cognitive[tiab] OR cognition[tiab] OR psychoeducat*[tiab] OR interpersonal[tiab] OR problem-solv*[tiab] OR network*[tiab] OR family[tiab] OR families[tiab] OR community[tiab] OR communities[tiab] OR group[tiab] OR groups[tiab] OR peer[tiab] OR self-help[tiab] OR selfhelp[tiab]** | [7,078,292](https://pubmed.ncbi.nlm.nih.gov/?term=psychology%5Btiab%5D+OR+psychological%5Btiab%5D+OR+psychosocial%5Btiab%5D+OR+psychotherapy%5Btiab%5D+OR+socio%2A%5Btiab%5D+OR+social%2A%5Btiab%5D+OR+educat%2A%5Btiab%5D+OR+behavio%2A%5Btiab%5D+OR+cope%5Btiab%5D+OR+coping%5Btiab%5D+OR+cognitive%5Btiab%5D+OR+cognition%5Btiab%5D+OR+psychoeducat%2A%5Btiab%5D+OR+interpersonal%5Btiab%5D+OR+problem-solv%2A%5Btiab%5D+OR+network%2A%5Btiab%5D+OR+family%5Btiab%5D+OR+families%5Btiab%5D+OR+community%5Btiab%5D+OR+communities%5Btiab%5D+OR+group%5Btiab%5D+OR+groups%5Btiab%5D+OR+peer%5Btiab%5D+OR+self-help%5Btiab%5D+OR+selfhelp%5Btiab%5D&sort=relevance&size=200&ac=no) |
| #4 | Search: **intervention*[tiab] OR therap*[tiab] OR program*[tiab] OR support[tiab] OR strateg*[tiab] OR skill*[tiab] OR counsel*[tiab] OR technique*[tiab]** | [7,003,563](https://pubmed.ncbi.nlm.nih.gov/?term=intervention%2A%5Btiab%5D+OR+therap%2A%5Btiab%5D+OR+program%2A%5Btiab%5D+OR+support%5Btiab%5D+OR+strateg%2A%5Btiab%5D+OR+skill%2A%5Btiab%5D+OR+counsel%2A%5Btiab%5D+OR+technique%2A%5Btiab%5D&sort=relevance&size=200&ac=no) |
| #3 | Search: **psychosocial support systems[mh] OR social support[mh] OR self-help groups[mh] OR psychotherapy[mh] OR counseling[mh] OR cognitive behavioral therapy[mh] OR adaptation, psychological[mh] OR health education/methods[mh] OR behavior therapy/methods[mh] OR mindfulness[mh]** | [448,600](https://pubmed.ncbi.nlm.nih.gov/?term=psychosocial+support+systems%5Bmh%5D+OR+social+support%5Bmh%5D+OR+self-help+groups%5Bmh%5D+OR+psychotherapy%5Bmh%5D+OR+counselling%5Bmh%5D+OR+cognitive+behavioral+therapy%5Bmh%5D+OR+adaptation%2C+psychological%5Bmh%5D+OR+health+education%2Fmethods%5Bmh%5D+OR+behavior+therapy%2Fmethods%5Bmh%5D+OR+mindfulness%5Bmh%5D&sort=relevance&size=200&ac=no) |
| #2 | Search: **adolescent[mh] OR adolescen*[tiab] OR teen[tiab] OR teens[tiab] OR teenage[tiab] OR teenager*[tiab] OR youth*[tiab] OR young adult[mh] OR young adult*[tiab] OR young person*[tiab] OR young people[tiab] OR young women[tiab] OR young females[tiab] OR young men[tiab] OR young males[tiab] OR youngster*[tiab] OR juvenil*[tiab] OR student*[tiab] OR pupils[tiab] OR young adulthood[tiab]** | [2,828,027](https://pubmed.ncbi.nlm.nih.gov/?term=adolescent%5Bmh%5D+OR+adolescen%2A%5Btiab%5D+OR+teen%5Btiab%5D+OR+teens%5Btiab%5D+OR+teenage%5Btiab%5D+OR+teenager%2A%5Btiab%5D+OR+youth%2A%5Btiab%5D+OR+young+adult%5Bmh%5D+OR+young+adult%2A%5Btiab%5D+OR+young+person%2A%5Btiab%5D+OR+young+people%5Btiab%5D+OR+young+women%5Btiab%5D+OR+young+females%5Btiab%5D+OR+young+men%5Btiab%5D+OR+young+males%5Btiab%5D+OR+youngster%2A%5Btiab%5D+OR+juvenil%2A%5Btiab%5D+OR+student%2A%5Btiab%5D+OR+pupils%5Btiab%5D+OR+young+adulthood%5Btiab%5D&sort=relevance&size=200&ac=no) |
| #1 | Search: **HIV Infections[MeSH] OR HIV[MeSH] OR hiv[tiab] OR hiv-1*[tiab] OR hiv-2*[tiab] OR hiv1[tiab] OR hiv2[tiab] OR hiv infect*[tiab] OR human immunodeficiency virus[tiab] OR human immunedeficiency virus[tiab] OR human immuno-deficiency virus[tiab] OR human immune-deficiency virus[tiab] OR ((human immun*[tiab]) AND (deficiency virus[tiab])) OR acquired immunodeficiency syndrome[tiab] OR acquired immunedeficiency syndrome[tiab] OR acquired immuno-deficiency syndrome[tiab] OR acquired immune-deficiency syndrome[tiab] OR ((acquired immun*[tiab]) AND (deficiency syndrome[tiab])) OR "sexually transmitted diseases, Viral"[MeSH:NoExp]** | [405,606](https://pubmed.ncbi.nlm.nih.gov/?term=HIV+Infections%5BMeSH%5D+OR+HIV%5BMeSH%5D+OR+hiv%5Btiab%5D+OR+hiv-1%2A%5Btiab%5D+OR+hiv-2%2A%5Btiab%5D+OR+hiv1%5Btiab%5D+OR+hiv2%5Btiab%5D+OR+hiv+infect%2A%5Btiab%5D+OR+human+immunodeficiency+virus%5Btiab%5D+OR+human+immunedeficiency+virus%5Btiab%5D+OR+human+immuno-deficiency+virus%5Btiab%5D+OR+human+immune-deficiency+virus%5Btiab%5D+OR+%28%28human+immun%2A%5Btiab%5D%29+AND+%28deficiency+virus%5Btiab%5D%29%29+OR+acquired+immunodeficiency+syndrome%5Btiab%5D+OR+acquired+immunedeficiency+syndrome%5Btiab%5D+OR+acquired+immuno-deficiency+syndrome%5Btiab%5D+OR+acquired+immune-deficiency+syndrome%5Btiab%5D+OR+%28%28acquired+immun%2A%5Btiab%5D%29+AND+%28deficiency+syndrome%5Btiab%5D%29%29+OR+%22sexually+transmitted+diseases%2C+Viral%22%5BMeSH%3ANoExp%5D&sort=relevance&size=200&ac=no) |

**Database: PsycINFO, via OVID**

**Date: 1 July 2020**

| 1 | ("human immunodeficiency virus infection" or "human immunodeficiency virus" or "human immuno deficiency virus" or "human immunedeficiency virus" or "human immune deficiency virus" or hiv or "hiv-1" or "hiv-2" or "acquired immunodeficiency syndrome" or "acquired immuno deficiency syndrome" or "acquired immunedeficiency syndrome" or "acquired immune deficiency syndrome").ti,ab,sh. | 54270 |  | |  |  |
| --- | --- | --- | --- | --- | --- | --- |
| 2 | (adolescen* or teen or teens or teenage or teenager* or youth* or "young adult*" or "young person*" or "young people" or "young women" or "young females" or "young men" or "young males" or youngster* or juvenil* or student* or pupils or "young adulthood").ti,ab,sh. | 853606 | |  |  |  |
| 3 | ("psychosocial support" or "social support" or "self help" or psychotherapy or counselling or counseling or "cognitive behavioral therapy" or "adaptive behavior" or "health education" or "behavior therapy" or mindfulness or "psychological aspect").ti,ab,sh. | 304747 | |  |  |  |
| 4 | (psychology or psychological or psychosocial or socio* or social* or educat* or behavio* or cope or coping or cognitive or cognition or psychoeducat* or interpersonal or "problem-solv*" or network* or family or families or community or communities or group or groups or peer or "self-help" or selfhelp).ti,ab,sh. | 3109579 | |  |  |  |
| 5 | (intervention* or therap* or program* or support or strateg* or skill* or counsel* or technique*).ti,ab,sh. | 1774018 | |  |  |  |
| 6 | 4 and 5 | 1304203 | |  |  |  |
| 7 | 3 or 6 | 1390606 | |  |  |  |
| 8 | 1 and 2 and 7 | 5858 | |  |  |  |
| 9 | ("randomized controlled trial" or random* or trial or allocat* or factorial* or placebo* or assign* or volunteer* or "crossover procedure" or "double-blind procedure" or "single-blind procedure" or (doubl* adj3 blind*) or (singl* and blind*) or crossover* or "cross+over*" or (cross adj1 over*)).ti,ab,sh. | 403593 | |  |  |  |
| 10 | 8 and 9 | 873 | |  |  |  |
| 11 | limit 10 to yr="2000 - 2020" | 788 | |  | | |

**Database: Cochrane Central Register of Controlled Trials
 Issue 7 of 12, July 2020**

**Date: 1 July 2020**

| **ID** | **Search** | **Hits** |
| --- | --- | --- |
| #1 | MeSH descriptor: [HIV Infections] explode all trees | 12295 |
| #2 | MeSH descriptor: [HIV] explode all trees | 3007 |
| #3 | hiv:ti,ab,kw or hiv*:ti,ab,kw or (hiv near infect*):ti,ab,kw or “human immunodeficiency virus”:ti,ab,kw or “human immunedeficiency virus”:ti,ab,kw or “human immune-deficiency virus”:ti,ab,kw or “human immuno-deficiency virus”:ti,ab,kw or “human immune deficiency virus”:ti,ab,kw or “human immuno deficiency virus”:ti,ab,kw or “acquired immunodeficiency syndrome”:ti,ab,kw or “acquired immunedeficiency syndrome”:ti,ab,kw or “acquired immuno-deficiency syndrome”:ti,ab,kw or “acquired immune-deficiency syndrome”:ti,ab,kw or “acquired immune deficiency syndrome”:ti,ab,kw or “acquired immuno deficiency syndrome”:ti,ab,kw (Word variations have been searched) | 27229 |
| #4 | MeSH descriptor: [Lymphoma, AIDS-Related] this term only | 21 |
| #5 | MeSH descriptor: [Sexually Transmitted Diseases, Viral] this term only | 27 |
| #6 | #1 or #2 or #3 or #4 or #5 | 27352 |
| #7 | [mh adolescent] or adolescen*:ti,ab,kw or teen:ti,ab,kw or teens:ti,ab,kw or teenage:ti,ab,kw or teenager*:ti,ab,kw or youth*:ti,ab,kw or [mh “young adult”] or “young adult”:ti,ab,kw or “young adults”:ti,ab,kw or “young person”:ti,ab,kw or “young persons”:ti,ab,kw or “young people”:ti,ab,kw or “young women”:ti,ab,kw or “young females”:ti,ab,kw or “young men”:ti,ab,kw or “young males”:ti,ab,kw or youngster:ti,ab,kw or youngsters:ti,ab,kw or juvenil*:ti,ab,kw OR student*:ti,ab,kw or pupils:ti,ab,kw or "young adulthood":ti,ab,kw (Word variations have been searched) | 213114 |
| #8 | [mh "psychosocial support systems"] or [mh "social support"] or [mh "self-help groups"] or [mh psychotherapy] or [mh counseling] or [mh "cognitive behavioral therapy"] or [mh "adaptation, psychological"] or [mh "health education"/MT] or [mh "behavior therapy"/MT] or [mh mindfulness] | 39085 |
| #9 | intervention*:ti,ab,kw or therap*:ti,ab,kw or program*:ti,ab,kw or support:ti,ab,kw or strateg*:ti,ab,kw or skill*:ti,ab,kw or counsel*:ti,ab,kw or technique*:ti,ab,kw (Word variations have been searched) | 1067495 |
| #10 | psychology:ti,ab,kw or psychological:ti,ab,kw or psychosocial:ti,ab,kw or psychotherapy:ti,ab,kw or socio*:ti,ab,kw or social*:ti,ab,kw or educat*:ti,ab,kw or behavio*:ti,ab,kw or cope:ti,ab,kw or coping:ti,ab,kw or cognitive:ti,ab,kw or cognition:ti,ab,kw or psychoeducat*:ti,ab,kw or interpersonal:ti,ab,kw or (problem next solv*):ti,ab,kw or network*:ti,ab,kw or family:ti,ab,kw or families:ti,ab,kw or community:ti,ab,kw or communities:ti,ab,kw or group:ti,ab,kw or groups:ti,ab,kw or peer:ti,ab,kw or “selfhelp”:ti,ab,kw or “self help”:ti,ab,kw (Word variations have been searched) | 873530 |
| #11 | #9 and #10 | 679007 |
| #12 | #8 or #11 | 681422 |
| #13 | #6 and #7 and #12 with Publication Year from 2000 to 2020, in Trials | 3290 |
