# Supplementary material for: Psychosocial interventions for improving engagement in care and health and behavioural outcomes for adolescents and young people living with HIV: a systematic review and meta‐analysis
Source: J Int AIDS Soc. 2021 Aug 2;24(8):e25741. doi: 10.1002/jia2.25741 (PMC8327356; doi:10.1002/jia2.25741)
Supplement: Supplementary file 2 — Table S2. List of definitions and examples of operationalized terms [file JIA2-24-e25741-s002.docx]

**Additional File 2: List of definitions and examples of operationalised terms**

|  | Term | Definition/examples of operationalized terms |
| --- | --- | --- |
| Population | Adolescents and young people living with HIV/AIDS | Adolescents and young people ages 10-24 with HIV diagnosis |
| Intervention | Psychosocial interventions | Psychosocial interventions are interpersonal or informational activities, techniques, or strategies that target behavioural, psychological, interpersonal, social factors with the aim of improving health and well-being.  e.g. peer support/networking, group-based life skills/social and emotional learning programmes etc. |
| Comparator | Care as usual/no intervention | Care as usual/no intervention, including medical care |

| Outcomes | Term | Definition/measurement/examples of operationalized terms |
| --- | --- | --- |
| Adherence to ART | Improved adherence to antiretroviral treatment | Reported adherence, viral load, CD4 count, pharmacy adherence measures i.e. medication possession ratio (MPR), pill count (PC), and pill pick-up (PPU), tablet counts, and electronic monitoring measures of adherence |
| ART knowledge | Improved knowledge about ART | Knowledge about ART, knowledge about HIV disease |
| Linkage to care | Number of days until linkage to ART after HIV diagnosis | Within 14 days, 30 days, or 90 days of HIV diagnosis (WHO definition); time to initiation |
| Retention in care | Regular engagement with medical care at a health care facility after initial entry into the system at regularly defined intervals | Attrition in care, missed visits/appointments, medical visits attended, appointment adherence (completed visits/scheduled visits), missed visit rate (no show visits/scheduled visits), visit consistency, gaps in care (e.g. more than 28 days, 3 months, and 6 months between visits) |
| Viral load | Reduction of viral load | Number of copies of HIV RNA in a millilitre of blood |
| Viral suppression | Suppression of viral load | Copies of HIV RNA in a millilitre of blood under threshold of 1000 copies/mL |
| Undetectable viral load | Undetectable viral load | Copies of HIV RNA in a millilitre of blood under threshold of 200 copies/mL |
| Sexual and reproductive health behaviours | Risky sexual and reproductive health behaviours | Condom use, early sexual debut, multiple partners, concurrent partners, contraception use, unintended pregnancy, transactional sex, age-disparate sex, sex while drunk or on drugs, sexually transmitted infections |
| Sexual and reproductive health knowledge | Improved knowledge about sexual and reproductive health | Condom use efficacy, motivation to use condoms, self-efficacy for disclosure, contraceptive knowledge, self-efficacy for limiting risk behaviour |
| Improved transitioning to adult services | Purposeful, planned movement from paediatric, child-centred or specialised adolescent services to adult-oriented services | Transition readiness, transition planning, transfer of care, transfer completion |
