# Supplementary figures and images for: Psychosocial interventions for improving engagement in care and health and behavioural outcomes for adolescents and young people living with HIV: a systematic review and meta‐analysis
Source: J Int AIDS Soc. 2021 Aug 2;24(8):e25741. doi: 10.1002/jia2.25741 (PMC8327356; doi:10.1002/jia2.25741)

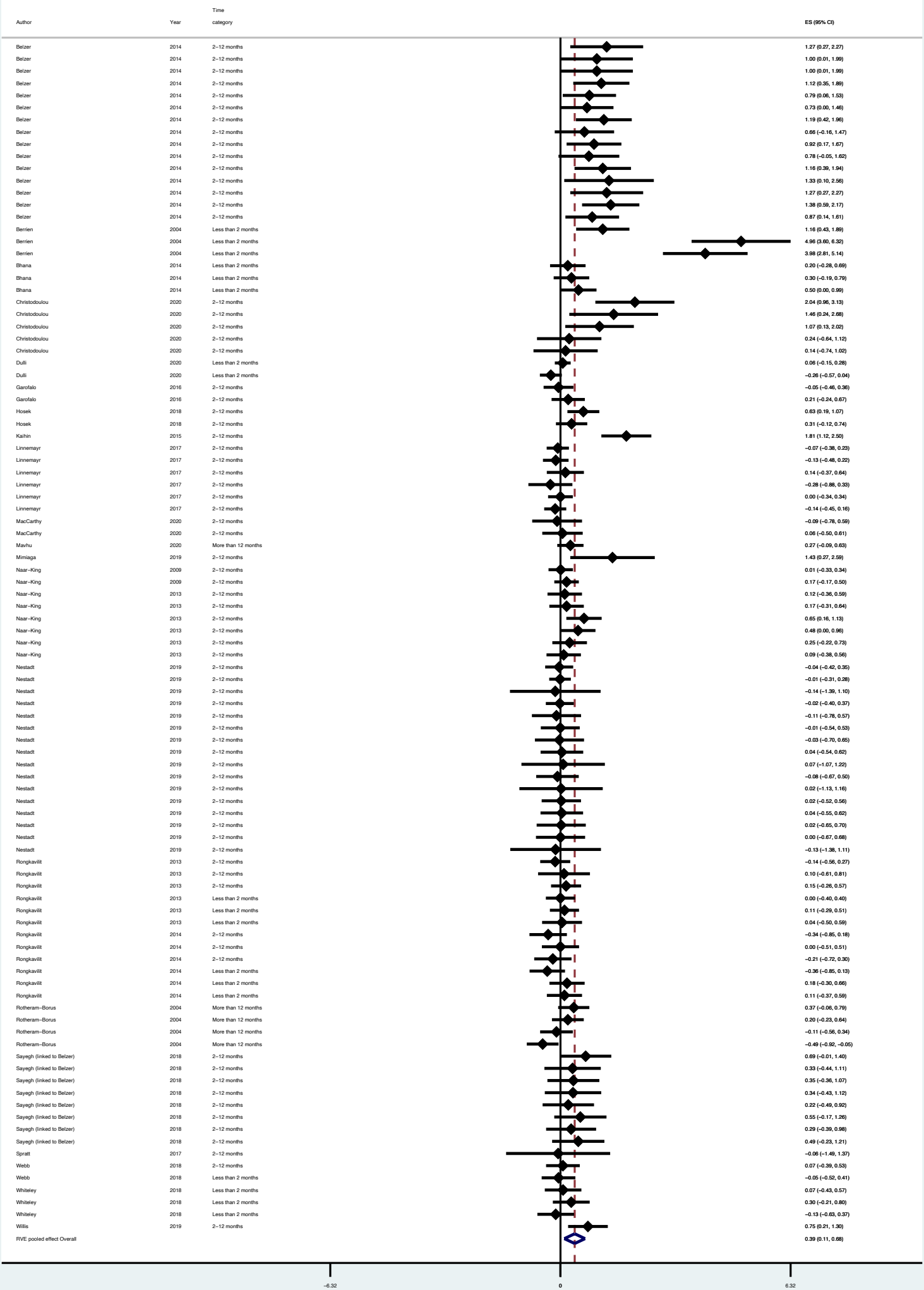

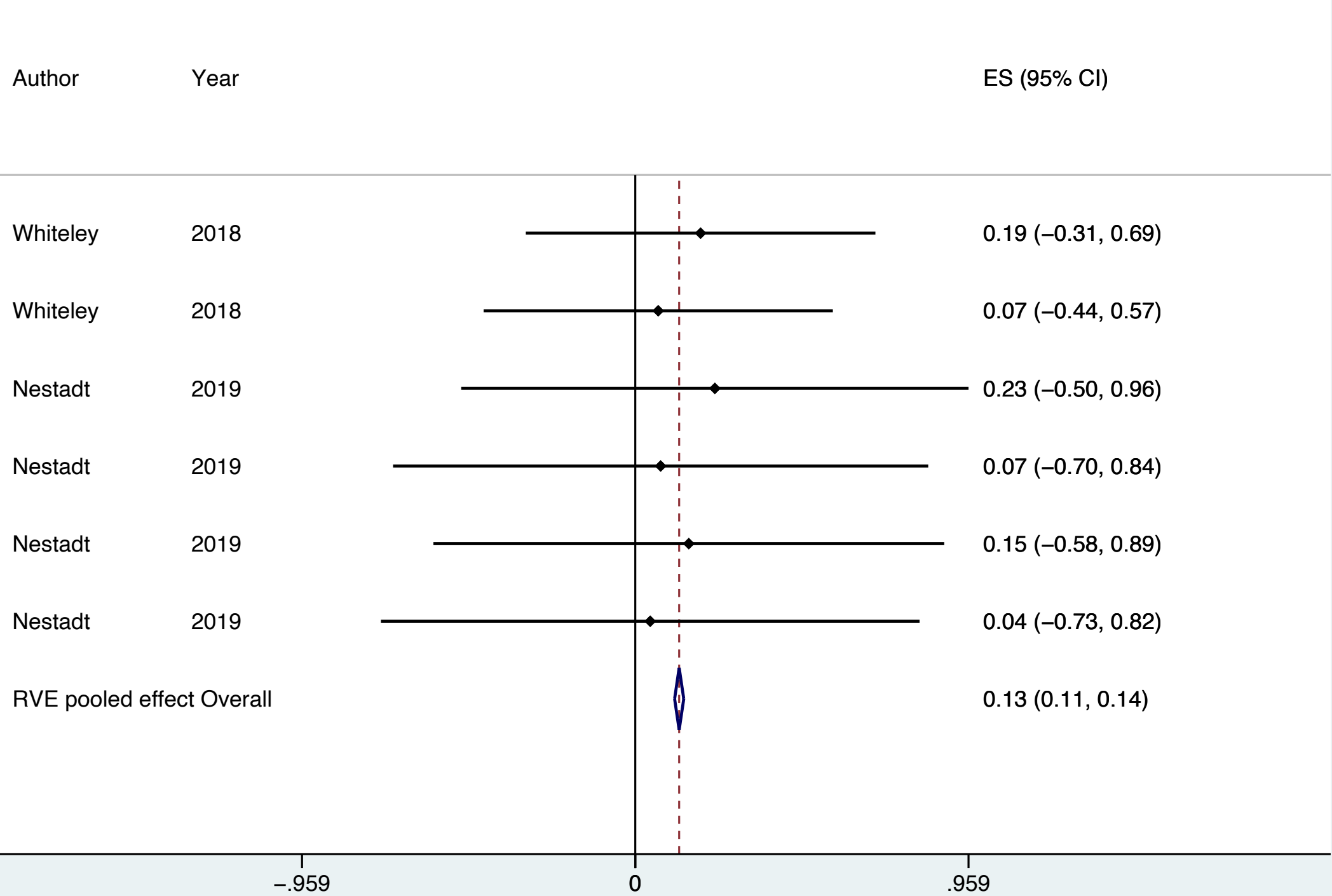

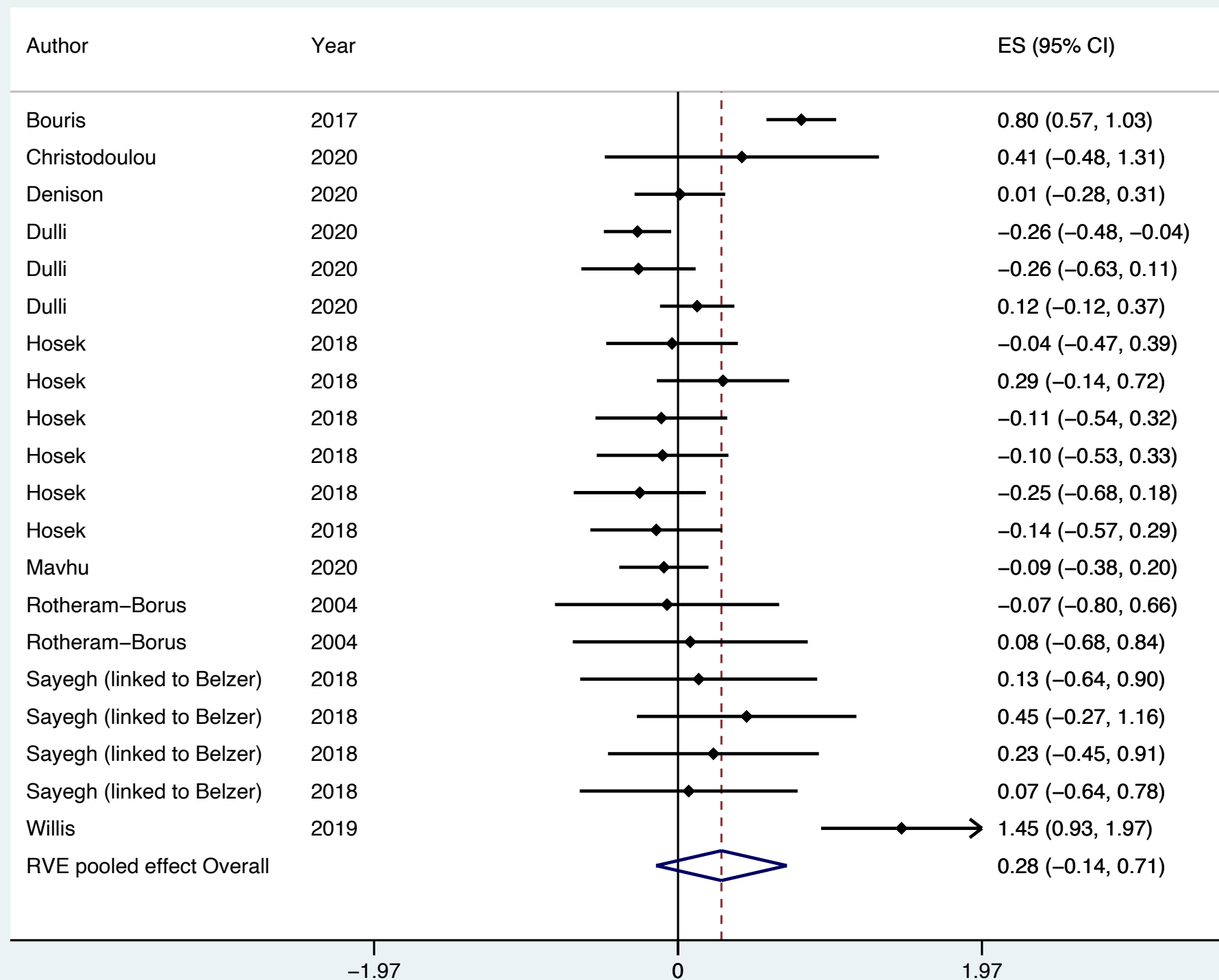

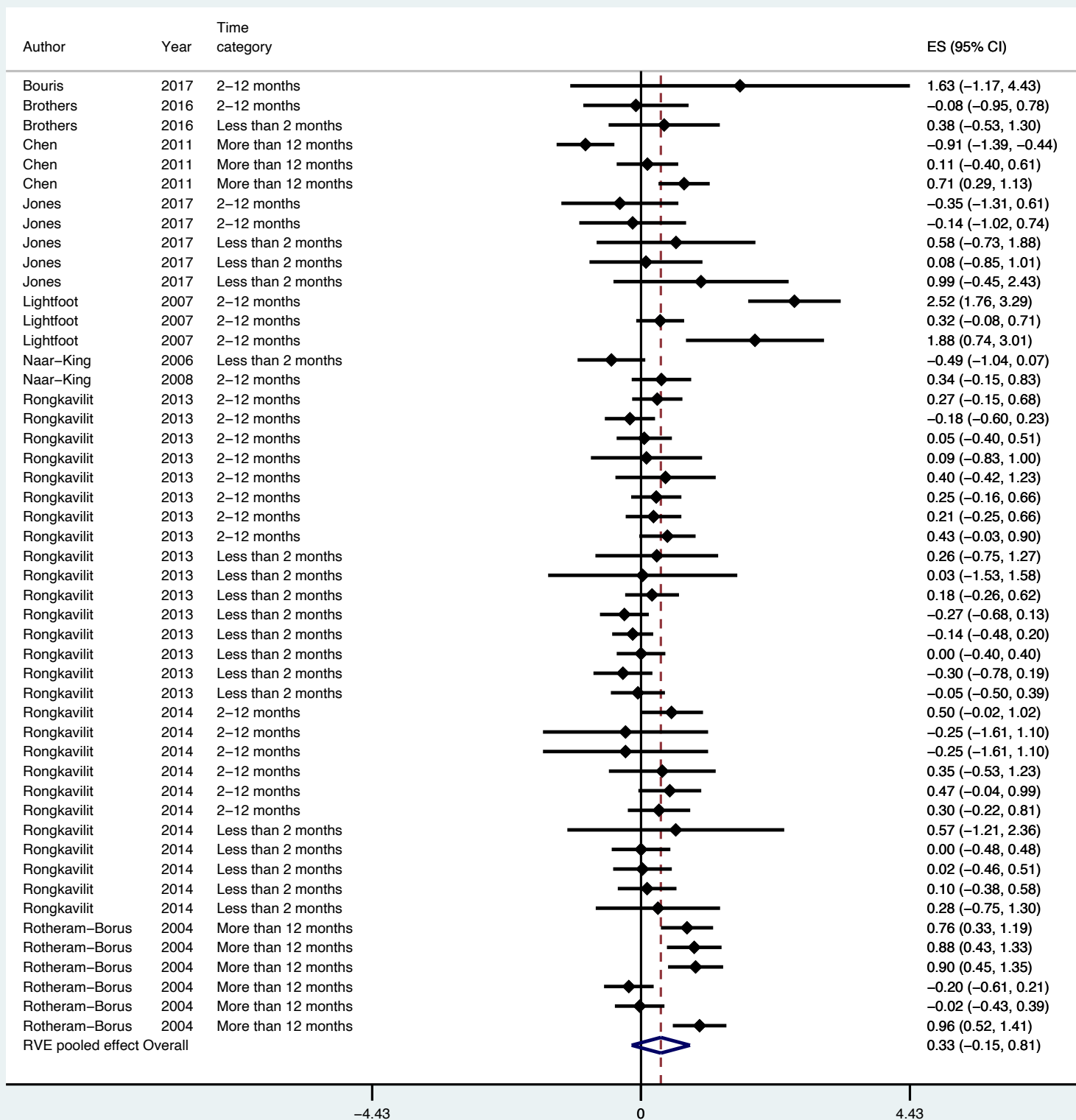

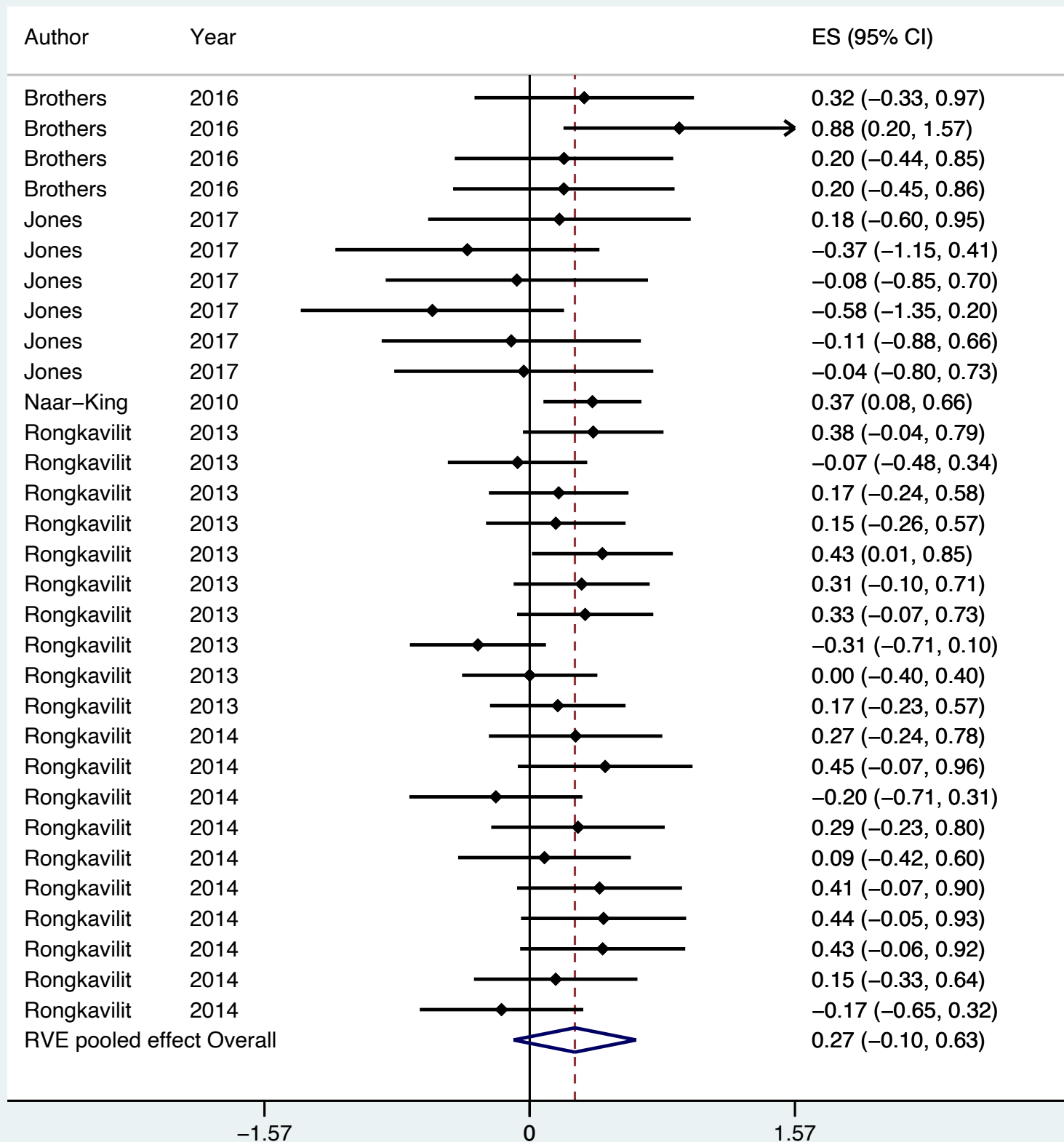

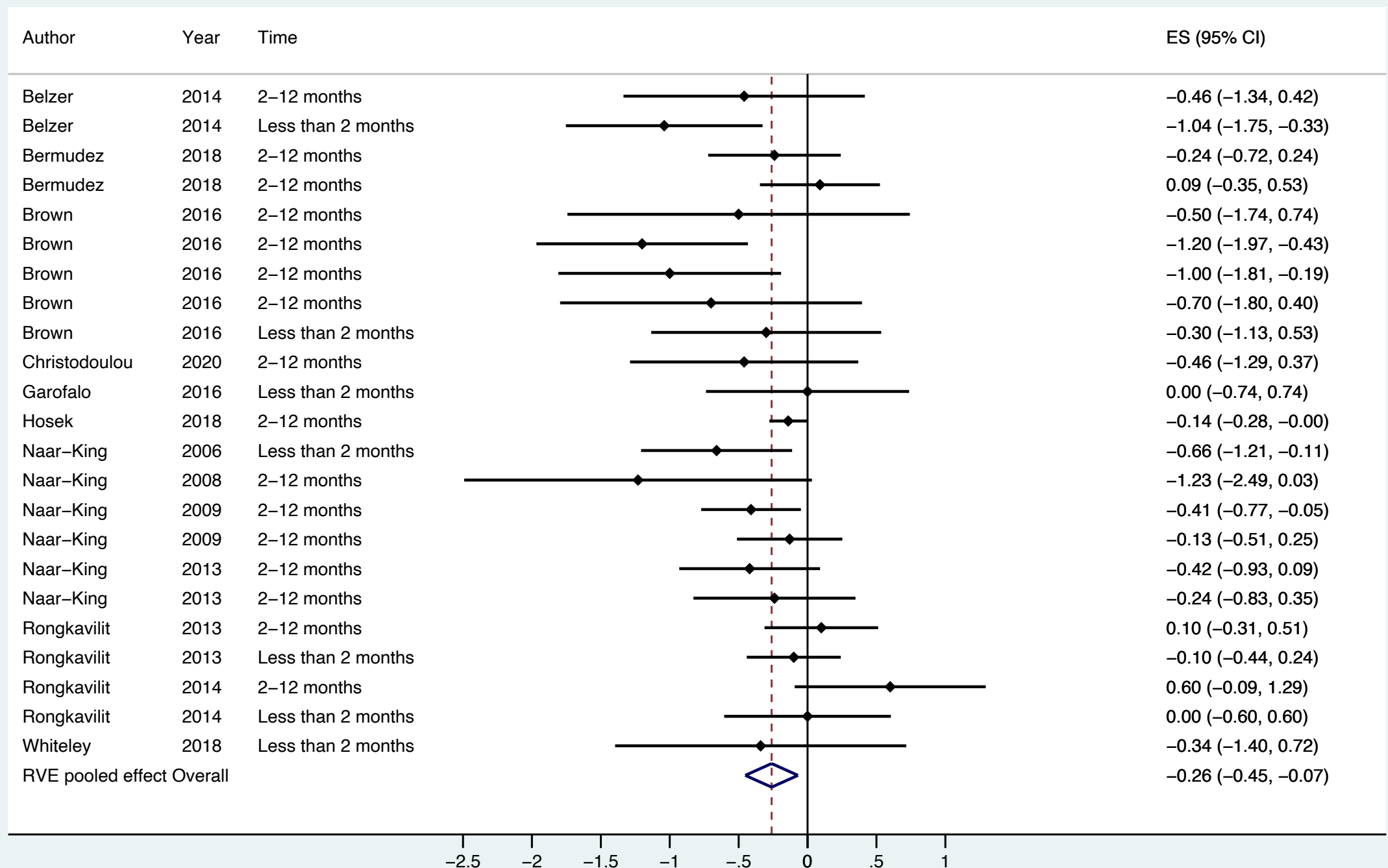

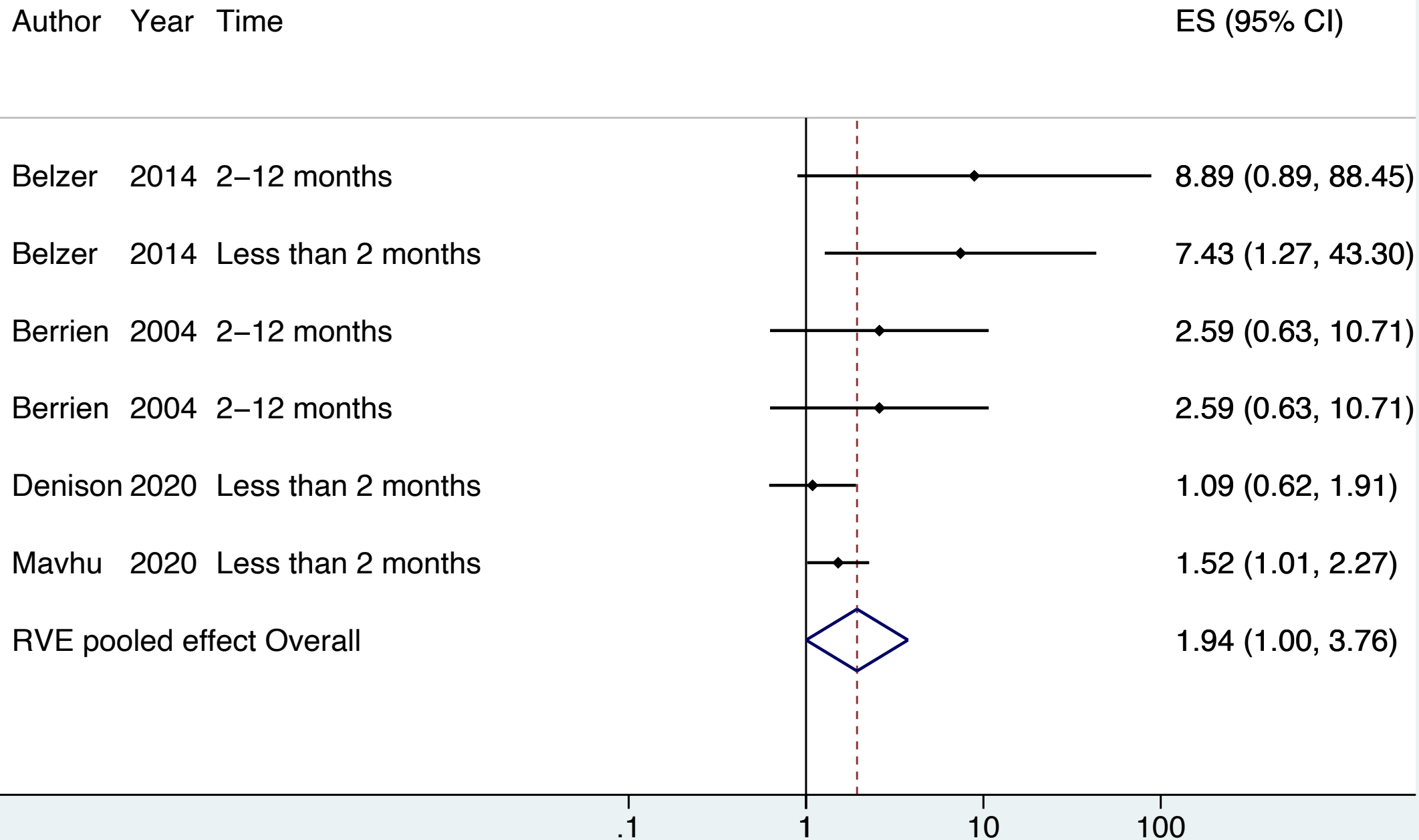

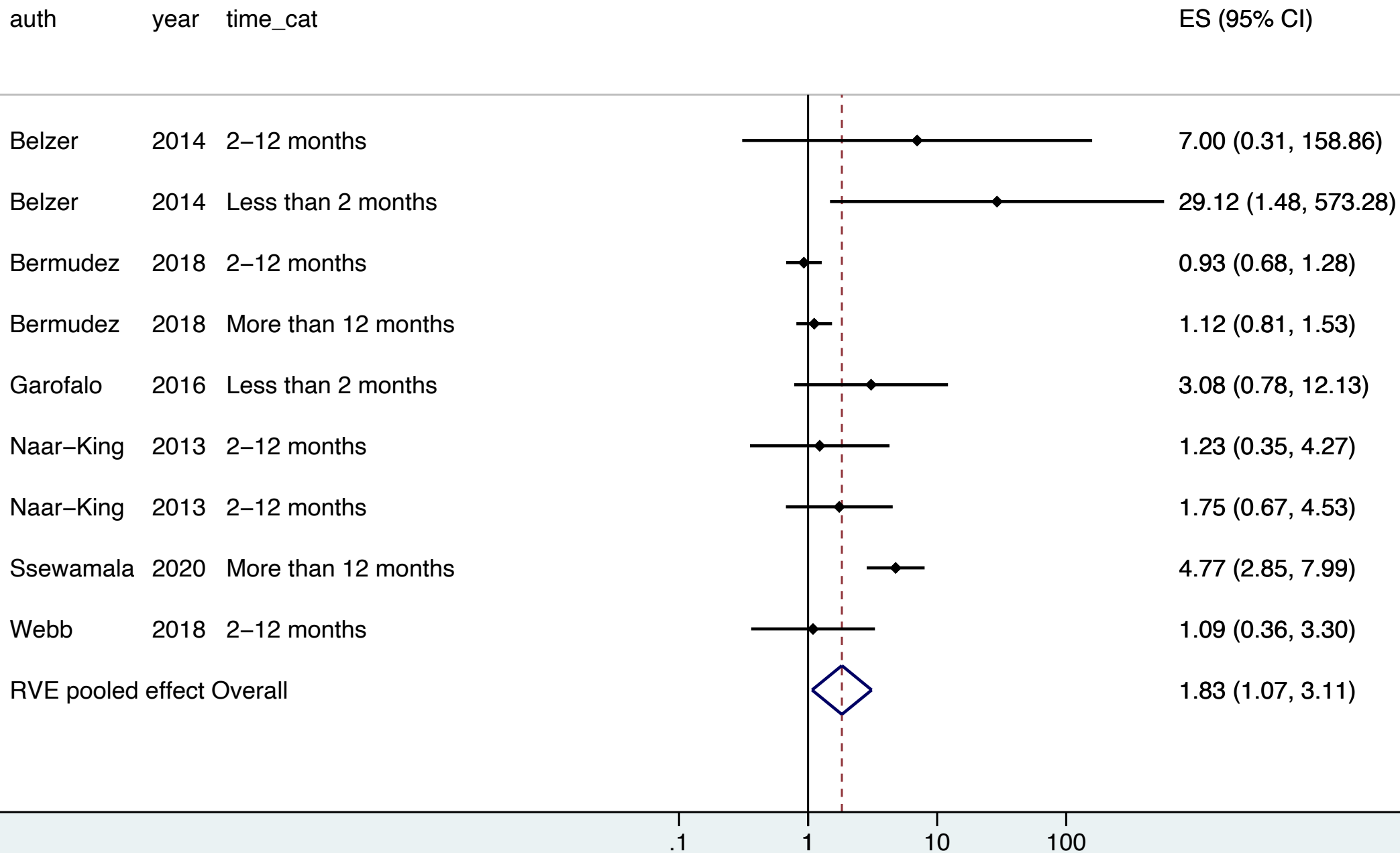

Supplement: Supplementary file 3 — Table S3. Forest plots for all outcomes [file JIA2-24-e25741-s001.pdf]

**Additional File 3: Risk of bias ratings**

**
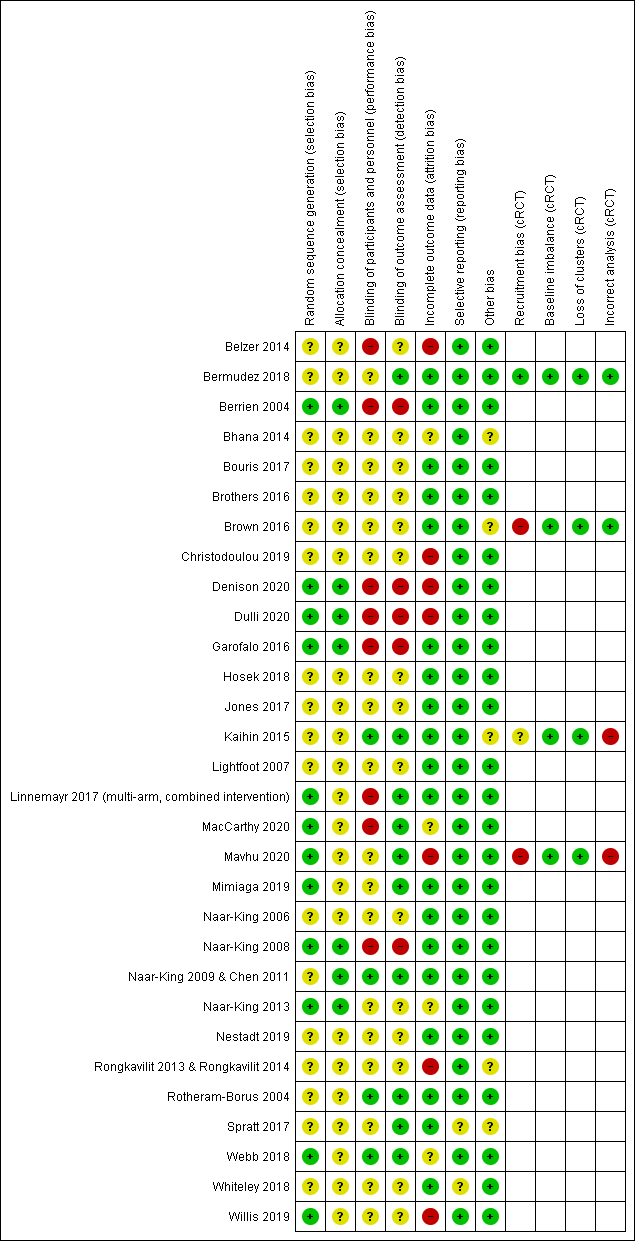
**

Supplement: Supplementary file 4 — Figure S1. Risk of bias diagram [file JIA2-24-e25741-s004.docx]
